# Regulation of anthocyanin accumulation via MYB75/HAT1/TPL-mediated transcriptional repression

**DOI:** 10.1371/journal.pgen.1007993

**Published:** 2019-03-15

**Authors:** Ting Zheng, Wenrong Tan, Huan Yang, Li’e Zhang, Taotao Li, Baohui Liu, Dawei Zhang, Honghui Lin

**Affiliations:** 1 Key Laboratory of Bio-Resource and Eco-Environment of Ministry of Education, College of Life Sciences, Sichuan University, Chengdu, Sichuan, P.R.China; 2 School of Life Science, Guangzhou University, Guangzhou, P.R.China; Peking University, CHINA

## Abstract

Anthocyanin is part of secondary metabolites, which is induced by environmental stimuli and developmental signals, such as high light and sucrose. Anthocyanin accumulation is activated by the MYB-bHLH-WD40 (MBW) protein complex in plants. But the evidence of how plants maintain anthocyanin in response to signals is lacking. Here we perform molecular and genetic evidence to display that HAT1 plays a new breaker of anthocyanin accumulation via post-translational regulations of MBW protein complex. Loss of function of HAT1 in the Arabidopsis seedlings exhibits increased anthocyanin accumulation, whereas overexpression of HAT1 significantly repressed anthocyanin accumulation. We found that HAT1 interacted with MYB75 and thereby interfered with MBW protein complex. Overexpression of HAT1 suppresses abundant anthocyanin phenotype of *pap1-D* plant. HAT1 is characterized as a transcriptional repressor possessing an N-terminal EAR motif, which determines to interact with TOPLESS corepressor. Repression activity of HAT1 in regulation of gene expression and anthocyanin accumulation can be abolished by deletion or mutation of the EAR motif 1. Chromatin immunoprecipitation assays revealed that MYB75 formed a transcriptional repressor complex with HAT1-TPL by histone H3 deacetylation in target genes. We proposed that HAT1 restrained anthocyanin accumulation by inhibiting the activities of MBW protein complex through blocking the formation of MBW protein complex and recruiting the TPL corepressor to epigenetically modulate the anthocyanin late biosynthetic genes (LBGs).

## Author summary

Anthocyanins, a class of flavonoids distributed ubiquitously in the plant kingdom, are induced by environmental stimuli and developmental signals, such as high light and sucrose. It is well established that anthocyanin accumulation is regulated by the MYB-bHLH-WD40 (MBW) protein complex in plants. But little is known about the regulation of MBW protein complex by other factors. Here, we show that an HD-ZIP II transcription factor HAT1 negatively regulates anthocyanin accumulation via post-translational regulation of MBW protein complex. Loss of function of HAT1 in the Arabidopsis seedlings exhibits increased anthocyanin accumulation, whereas overexpression of HAT1 significantly repressed anthocyanin accumulation. We reveal that HAT1 interacted with MYB75 and thereby sequestered MBW protein complex. Overexpression of HAT1 in *pap1-D* mutant suppresses abundant anthocyanin phenotype of the *pap1-D* mutant. HAT1 identified was as a transcriptional repressor possessing an N-terminal EAR motif, which determines the interaction with TOPLESS corepressor. The deletion or mutation of the EAR motif 1 of HAT1 partially eliminates the repression activity of HAT1 in regulation of gene expression and anthocyanin accumulation. Our results illustrate a new repressor HAT1 which helps plants fine-tune anthocyanin accumulation.

## Introduction

Anthocyanins, one kind of flavonoids, are vital secondary metabolites widespread throughout the plant kingdom [1]. As water-soluble pigments, anthocyanins confer widest colors to flowers, leaves, and fruits [2]. Anthocyanin accumulation is stimulated by a variety of endocellular signals such as sucrose and phytohormone [3, 4], as well as exogenous environmental stresses including high light [5], drought [6], and nutrient depletion [7]. Anthocyanins can protect plants against excessive light [8] and drought [9], and defend from invasion by pathogens and herbivores [10].

Anthocyanin biosynthesis is derived from flavonoid synthetic pathway which is composed of multiple enzymes encoded by biosynthetic genes. Initially, the early flavonoid reactions catalyzed by early biosynthetic genes (EBGs) included chalcone synthase (*CHS*), chalcone isomerase (*CHI*), and flavonol 3-hydroxylase (*F3H*), which are regulated by three redundant R2R3 MYB transcription factors (TFs) MYB11, MYB12, and MYB111 [11]. Then the expression of anthocyanin-specific biosynthetic genes encoding dihydroflavonol-4-reductase (*DFR*), leucoanthocyanidin dioxygenase (*LDOX*), and UDP-glucose: flavonoid-3-O-glycosyl-transferase (*UF3GT*) is regulated by the ternary MYB-bHLH-WD40 (MBW) protein complex, which is composed of R2R3-MYB, basic helix-loop-helix (bHLH), and WD40-repeat proteins [2, 12]. In Arabidopsis, the identified R2R3-MYB transcription factors include PRODUCTION OF ANTHOCYANIN PIGMENTATION 1 (PAP1)/MYB75, PAP2/MYB90, MYB113, and MYB114 [13, 14]. The bHLH transcription factors include TRANSPARENT TESTA 8 (TT8) and ENHANCER OF GLABRA 3 (EGL3), and and only one WD40-repeat protein, TRANSPARENT TESTA GLABRA 1 (TTG1), has been identified [14–16].

Notably, some MYB TFs such as TRANSPARENT TESTA 2 (TT2), GLABRA1 (GL1), and WEREWOLF (WER) act as a component of MBW protein complex to transcriptionally regulate expression of multiple gene involved in proanthocyanin accumulation, trichome development, and root epidermal cell fate in *Arabidopsis thaliana* [17–19]. Recent studies demonstrated that post-translational modification of MBW proteins modulated the transcriptional activity of MBW protein complex. Degradation of MYB75 in the dark was mediated by E3 ubiquitin ligase COP1 [20], while MPK4 phosphorates MYB75 and increases its stability in response to light [5]. Ubiquitin protein ligase 3 (UPL3) regulates anthocyanin and trichome development by mediating the proteasomal degradation of GL3 and EGL3 [21]. GSK3-like kinase BIN2 controls root epidermal cell fate through suppressing the activity of MBW protein complex via phosphorylating both TTG1 and EGL3 [22]. The other post-translational regulation is preventing the formation of MBW protein complex. Single repeat R3-MYB transcription factors, including MYBL2, CAPRICE (CPC), TRIPTYCHON (TRY), ENHANCER OF TRY AND CPC 1 (ETC1), and ETC2, suppress anthocyanin accumulation or trichome initiation by disturbing the formation of MBW protein complex [23–27]. SPL9, a member of SQUAMOSA PROMOTER BINDING PROTEIN-LIKE family, negatively regulates anthocyanin accumulation by directly preventing expression of anthocyanin biosynthetic genes via destabilization of MBW complex [28]. Similarly, the JA-ZIM domain (JAZ) proteins suppress anthocyanin accumulation and trichome development by disturbing the MBW protein complex [4]. Additionally, MYBL2 and *Ph*MYB27 transform the MBW complex from an activator to a repressor by replacing one of the R2R3-MYB components of MBW protein [23, 24, 29]. The transformation possibly depends on post-translational regulation. However, details of the regulation mechanism remain unclear.

Anthocyanin accumulation in plants is modulated by light conditions [20]. Plants accumulate less anthocyanin under shade conditions [29]. It is well established that the class II homeodomain-leucine zipper family participate in shade avoidance and their expression is rapidly induced by shade conditions [30]. The members of class II homeodomain-leucine zipper family all contain a conserved DNA-binding homeodomain (HD) that is closed to a leucine zipper motif (LZ), which is considered important to promote homo- or heterodimerization of HD-Zip protein [31, 32]. The CPSCE motif adjacent to LZ motif is comprised of five conserved amino acids Cys, Pro, Ser, Cys, and Glu. This motif is thought to form high molecular weight multimers through intermolecular Cys-Cys bridges under oxidant environment, which can not possibly be transported to the nucleus to play its role [33]. It was reported that the class II HD-Zip proteins participated in modulation of plant development and multiple stress response. HAT2 is strongly induced by auxin and affects lateral root and hypocotyl elongation [34]. HAT3 and ATHB4 impact the leaf polarity in *Arabidopsis thaliana* by repressing MIR165/166 expression [35]. ATHB4 and HAT1 participate in Brassinosteroid signaling [36, 37]. ATHB17 is involved in ABA response and plays an important role in protecting plants by adjusting expression of *PhANGs* and *PEGs* in response to abiotic stresses [38, 39].

To explore the regulatory mechanisms in anthocyanin accumulation, here we identify a new regulator of MBW protein complex. HAT1 interferes with the formation of MBW protein complex by interacting with MYB75. In Arabidopsis, TOPLESS (TPL) and TOPLESS RELATED (TPR) proteins generally mediate transcriptional repression in numerous pathways such as auxin and jasmonate signaling [40–42], as well as developmental pathways including leaf polarity and flowering time regulation [43, 44]. Meanwhile, we reveal that TPL can interact with HAT1. We propose that HAT1 represses anthocyanin accumulation by inhibiting the activities of MBW protein complex through recruiting the TPL/TPR corepressors and histone modification. Our study exposes that HAT1 acts as a new regulator of anthocyanin accumulation, and proffers a mechanism for repression of anthocyanin accumulation.

## Results

### Negative correlation of HAT1 levels and anthocyanin accumulation

**Fig 1:**
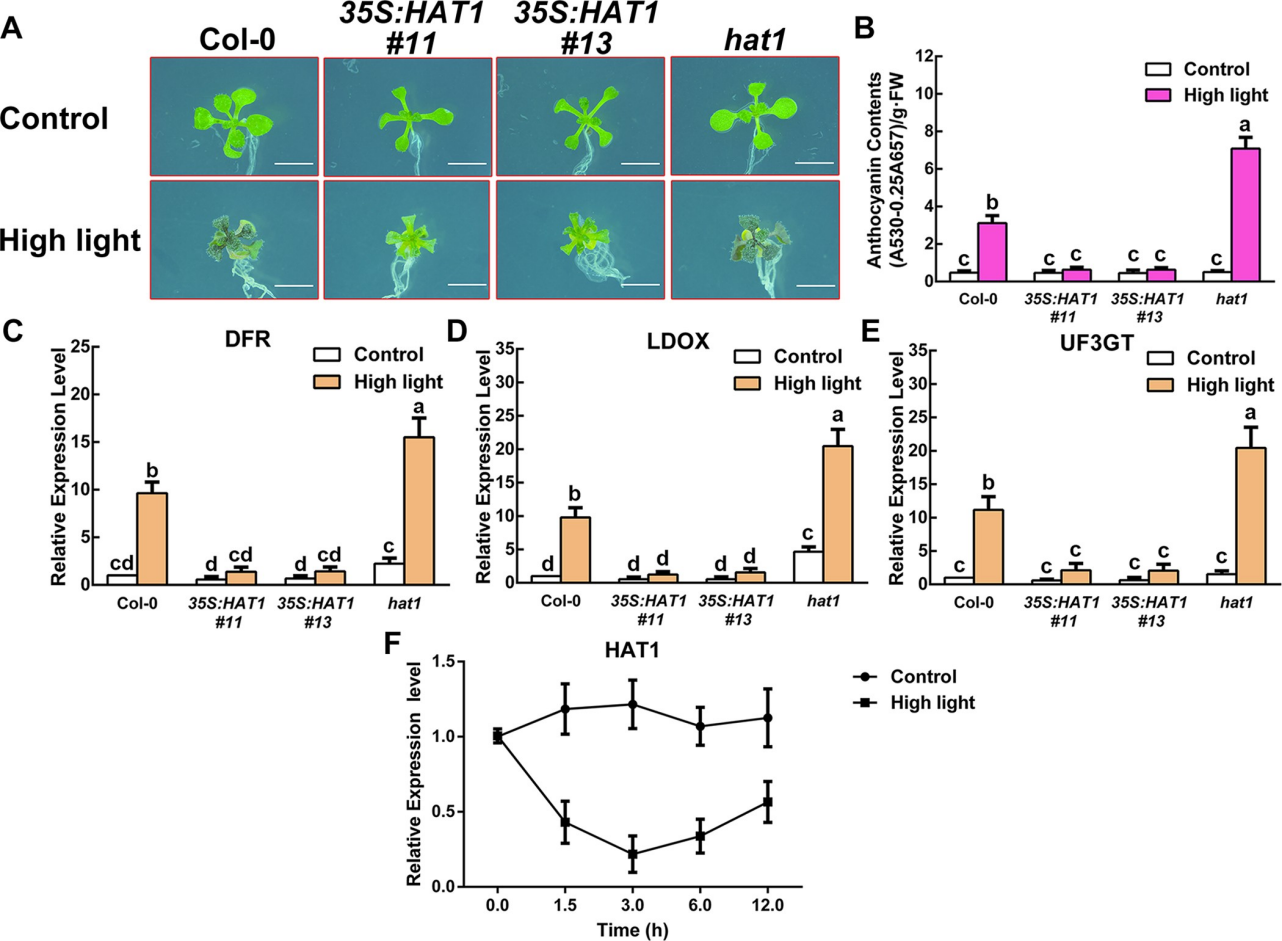
HAT1 repressed anthocyanin accumulation under high light conditions. (A) 14-day-old Arabidopsis seedlings of Col-0, *35S*:*HAT1 #11*, *35S*:*HAT1 #13*, *hat1* grown on plates under different conditions. Bars = 0.5 cm. (B) Anthocyanin levels in extracts from seedlings in (A). The experiments were performed in biological triplicate (representing anthocyanin content measured from 15 plants of each genotype and treatment were pooled for one replicate). FW, fresh weight. Error bars denote ± SD (n = 3). Different letters represented statistically significant differences (two-way ANOVA, p<0.05). (C-E) qPCR analysis of *DFR*, *LDOX*, and *UF3GT* expression levels in 14-day-old seedlings in (A) grown on plates under different conditions for 9 h, respectively. Expression levels were standardized to *ACTIN 8*, the results of Col-0 under control conditions were set at 1. Error bars denote ± SD (n = 3). Different letters represented statistically significant differences (two-way ANOVA, p<0.05). (F) HAT1 expression in Arabidopsis under control and high light conditions. The Col-0 plants were grown on plates under control conditions for 14 d and moved to high light conditions to collect seedlings at different time point for HAT1 transcript levels detection by qRT-PCR. Control is the same without light changes and expression levels of the HAT1 in the 0 h under control conditions were set at 1. Error bars denote ± SD (n = 3).

Our previous research has proved that HAT1 participated in drought response [45], and we noticed that transgenic plants overexpressing HAT1 (*35S*:*HAT1*) showed less anthocyanin accumulation compared with wild-type plants under drought stress. In Arabidopsis, the levels of anthocyanin accumulation are dependent on light intensity [46]. To understand how HAT1 influences anthocyanin accumulation, we maintained Arabidopsis seedlings under weak light of 40 μmol m^-2^ s^-1^ (hereafter called Control), a control light intensity at which wild-type plants accumulate very low levels of anthocyanins [47]. To induce anthocyanin biosynthesis, seedlings were then shifted to moderate high light (180 μmol m^-2^ s^-1^, hereafter called high light). Exposed to high light, two independent lines overexpressing HAT1 (*35S*:*HAT1 #11* and *#13*) accumulated less anthocyanin than that of wild-type plants, while *hat1* mutant showed higher anthocyanin accumulation (Fig 1A and 1B). We also detected the expression levels of anthocyanin-specific biosynthetic genes, *DFR*, *LDOX* and *UF3GT*. The expression of these genes was also reduced in *35S*:*HAT1* plants but induced in the *hat1* mutant (Fig 1C–1E). Additionally, We also noticed that HAT1 transcription was notably repressed under high light conditions (Fig 1F). *35S*:*HAT1 #13* transgenic plants were selected for subsequent experiment because the similar phenotype was observed between *35S*:*HAT1 #11* and *35S*:*HAT1 #13* transgenic plants. As shown in S1 Fig, *35S*:*HAT1 #13* transgenic plants grown in soil exhibited less anthocyanin accumulation compared with wild-type under high light conditions.

Sucrose can specifically induce anthocyanin biosynthesis in Arabidopsis, thus we further investigate the anthocyanin accumulation in Arabidopsis seedlings grown with exogenous sucrose [3]. Similarly, the anthocyanin content of *35S*:*HAT1 #13* was significantly lower than that of wild-type under sucrose treatment (S2A and S2B Fig). Compared with wild-type, *DFR* transcripts were decreased in *35S*:*HAT1 #13* but increased in *hat1* mutant. *LDOX* and *UF3GT* showed the similar expression pattern to that of *DFR* (S2C Fig). In Arabidopsis, some transcription factors modulate the expression of anthocyanin biosynthetic genes. We further examined the expression of several transcription factors including *MYB75*, *MYB90*, *TT8*, *EGL3* and *TTG1*, which are characterized as regulators of anthocyanin biosynthetic genes (S2D Fig). The expression levels of *MYB75* and *MYB90* obviously decreased in *35S*:*HAT1 #13* when compared with wild-type, while a nearly 1.5-fold increased was recorded in *hat1* mutant than that in the wild-type (S2D Fig). The transcript levels of *TT8* and *EGL3* were moderately increased in *hat1* mutant (S2D Fig). In summary, these results suggest that HAT1 may play as a negative regulator in anthocyanin biosynthetic pathway.

### HAT1 physically interacts with MYB75

**Fig 2:**
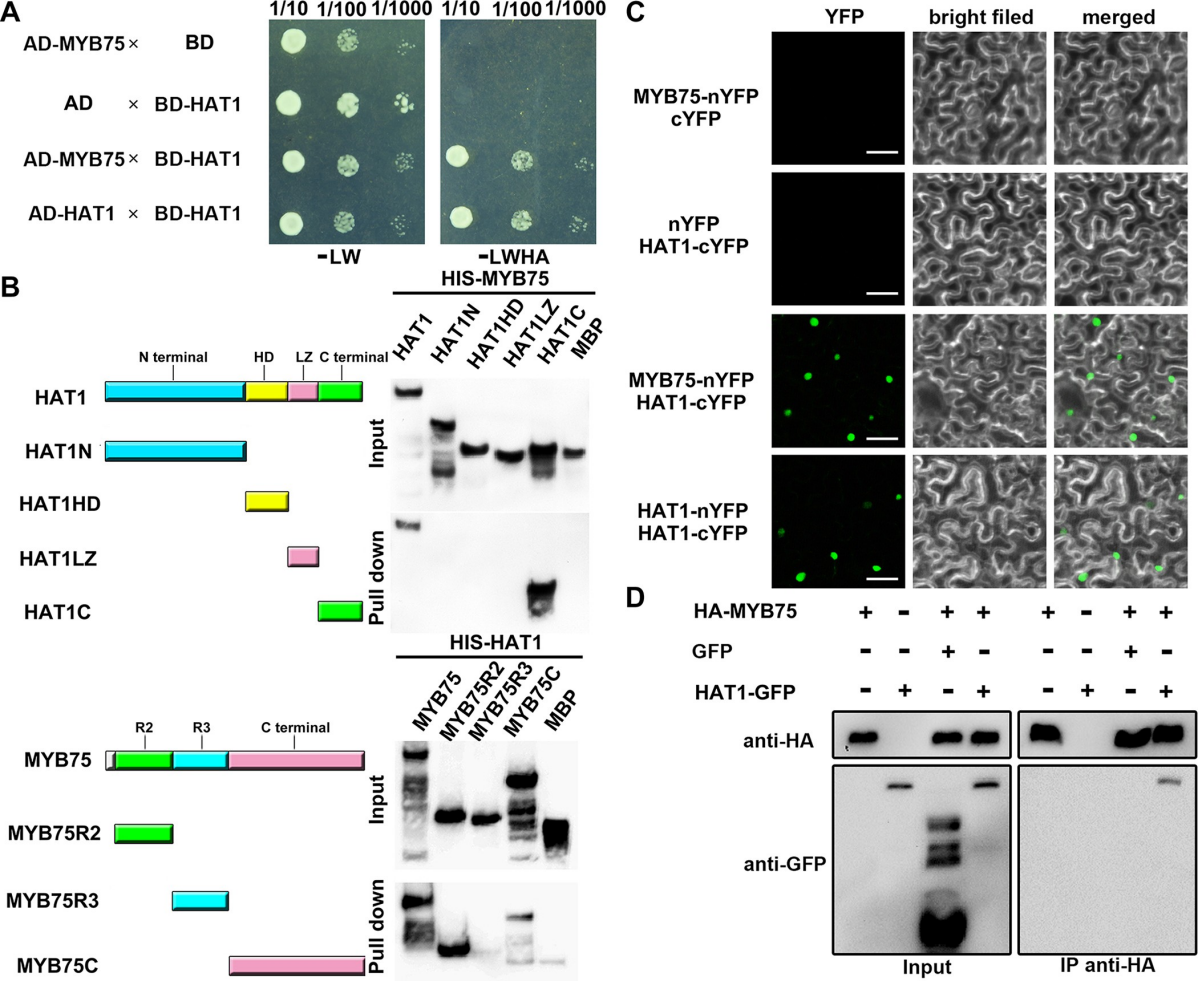
HAT1 interacts MYB75 *in vivo* and *in vitro*. (A) Yeast-two-hybrid assay. The ability of cells to grow on Quadruple DO supplement lacking Leu, Trp, His, and Ade (-LWHA) suggested the interaction. AD, GAL4 activation domain. BD, GAL4 DNA binding domain. (B) Pull-down assay. Full-length MYB75 interacts with different fragments of HAT1, including the full-length, N-terminus, HD domain, LZ domain, and C-terminus. Full-length HAT1 interacts with different fragments of MYB75, including the full-length, R2 domain, R3 domain, and C-terminus. (C) BiFC assay. HAT1 interacts with MYB75 in *N*. *benthamiana* leaves. Green indicates a positive interaction signal. No signal was observed from negative controls. Bars = 50 μm. (D) Co-IP analysis. Co-IP was performed using transgenic Arabidopsis plants expressing 35S:HA-MYB75, 35S:HAT1-GFP, co-expressing 35S:HA-MYB75 and 35S:GFP, or co-expressing 35S:HA-MYB75 and 35S:HAT1-GFP. HA-MYB75 and HAT1-GFP were perceived by anti-HA and anti-GFP antibodies, respectively.

To clarify how HAT1 regulates anthocyanin accumulation, we performed a yeast two-hybrid screen to identify its potential interaction partners. After screening, we identified MYB75 (At1g56650), an R2R3-MYB transcription factor, as its partner. Directed yeast two-hybrid assays validated that HAT1 interacted with MYB75, but not with MYB90, the paralog of MYB75 (Fig 2A, S3 Fig). To further determine the domains required for the interaction, truncated HAT1 and MYB75 were used. As shown in Fig 2B, C-terminal fragment of HAT1 was required for the interaction. The R2 domain of MYB75 could strongly interact with HAT1, but the C-terminal fragment of MYB75 weakly interacted with HAT1 (Fig 2B).

To verify whether HAT1 interacts with MYB75 *in vivo*, the bimolecular fluorescence complementation (BiFC) assay was performed for analysis. When MYB75-nYFP was coinfiltrated with HAT1-cYFP in tobacco (*Nicotiana benthamiana*) leaves, strong YFP fluorescence was observed in the nuclei (Fig 2C). Further, the interaction between HAT1 and MYB75 was also confirmed by a coimmunoprecipitation (Co-IP) assay (Fig 2D). These results suggest that HAT1 can interact with MYB75 *in vivo*.

### HAT1 interferes with the formation of MBW protein complex

**Fig 3:**
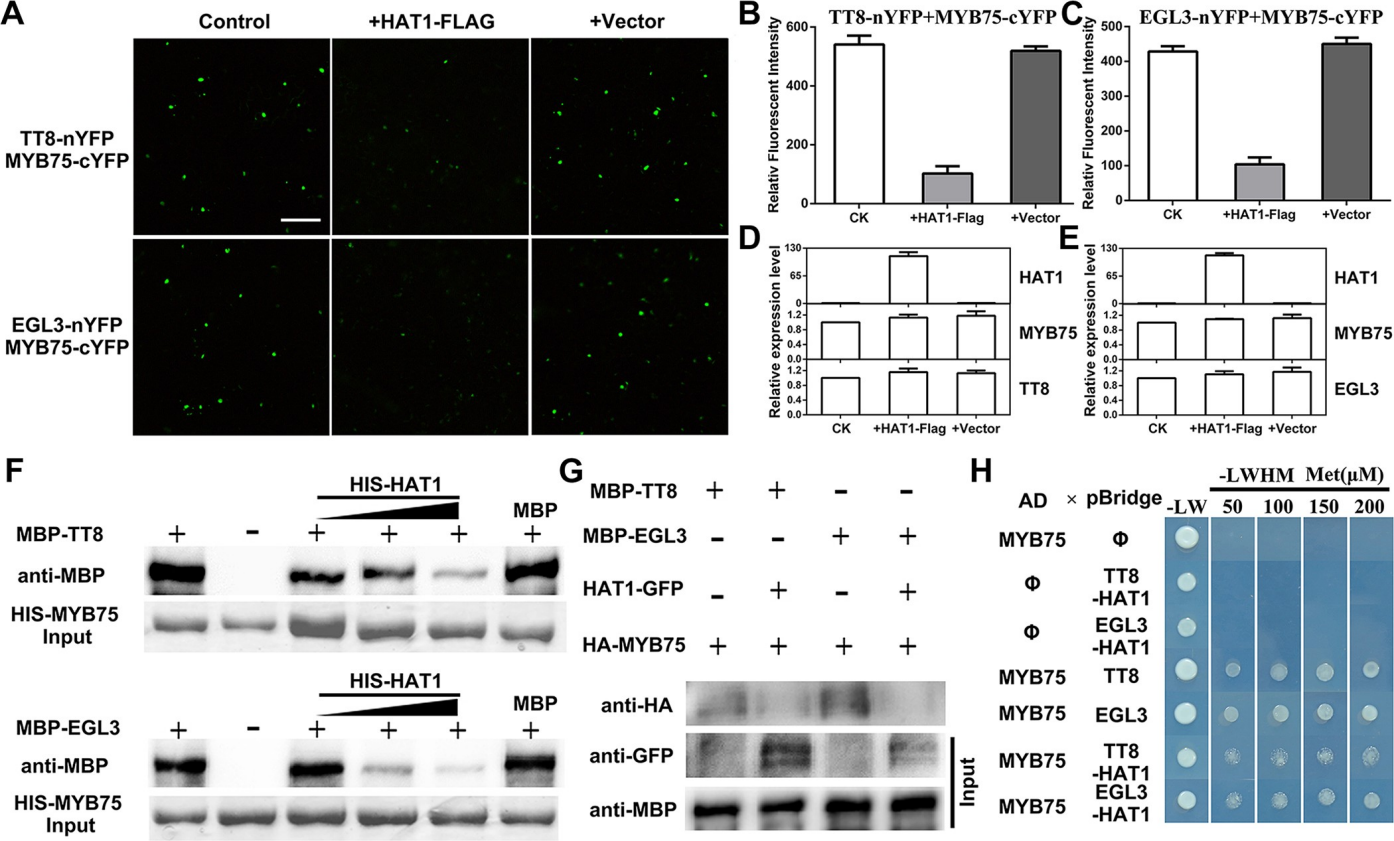
HAT1 inhibit the formation of MBW protein complex. (A) BiFC assays showing that HAT1 interferes the interaction of TT8 or EGL3 with MYB75. YFP fluorescence was observed 48 h after coexpression of TT8-nYFP or EGL3-nYFP/MYB75-cYFP (Control), HAT1-Flag/TT8-nYFP or EGL3-nYFP/MYB75-cYFP (+HAT1-Flag), or Flag/TT8-nYFP or EGL3-nYFP/MYB75-cYFP (+Vector). Bar = 100 μm. (B-C) Quantification of YFP fluorescence intensity in (A). Fifty separate fluorescent flecks were evaluated for fluorescence intensity. Error bars denote ± SD (n = 3). (D-E) qPCR analysis of *HAT1*, TT8-nYFP (*TT8*), EGL3-nYFP (*EGL3*) and MYB75-cYFP (*MYB75*) expression in (A). The primers used for amplificating Arabidopsis TT8-nYFP (*TT8*), EGL3-nYFP (*EGL3*) and MYB75-cYFP (*MYB75*) are shown in S1 Table online. Expression levels were standardized to *Nicotiana Actin*, and results of control treatment were set at 1. Error bars denote ± SD (n = 3). (F) Competitive binding assays of TT8 or EGL3 and HAT1 to MYB75. The gradient indicates the increasing amount of HIS-HAT1. The immunoprecipitated fractions were detected by anti-MBP antibody. HIS-HAT1 input is shown in the lower panel. (G) The effects of HAT1 on the MYB75-TT8 and MYB75-EGL3 interaction in plant cells. HA-MYB75 was expressed in protoplasts from wild-type or transgenic plants overexpressing HAT1-GFP with MBP-TT8 or MBP-EGL3. The interaction was detected by immunoprecipitation on anti-MBP resin. The immunoprecipitated proteins (IP) were detected with an antibody recognizing HA. The expression levels of HA-MYB75 and HAT1-GFP were also detected using the indicated antibodies. (H) HAT1 competes with TT8 or EGL3 for binding to MYB75 in yeast. Modulated by the Methionine (Met)-suppressive MET25 promoter, HAT1 was co-expressed with indicated AD and pBridge fusion proteins. Yeast cells grown on the media suppied with the indicated Met levels.

Previous evidence demonstrated that ternary MYB75-TT8/EGL3-TTG1 protein complex can activate the expression of LBGs [14]. The results above suggest that HAT1 interacts with MYB75 and represses the transcripts levels of LBGs, hence, we speculate that HAT1 competed with bHLH proteins for interaction with MYB75 since no interaction between HAT1 and TT8/EGL3 was detected by BiFC assay (S4 Fig). To test this hypothesis, TT8-nYFP and MYB75-cYFP were transiently coexpressed in *N*. *benthamiana* leaves. As shown in Fig 3A, the strong YFP fluorescence were detected in *N*. *benthamiana* leaves, which is consistent with the previous study [48]. When HAT1-FLAG was coexpressed with TT8-nYFP and MYB75-cYFP, the fluorescence signal was visibly impaired (Fig 3A and 3B), whereas coexpression of empty vector (FLAG) with TT8-nYFP/MYB75-cYFP did not reduce the fluorescence intensity. Similar results were observed when HAT1-FLAG was coexpressed with EGL3-nYFP and MYB75-cYFP (Fig 3A and 3C). The expression of MYB75, TT8 and EGL3 exhibited no obvious difference in these combination respectively (Fig 3D and 3E).

*In vitro* competitive binding assays demonstrated that interaction between HIS-MYB75 and MBP-TT8 was gradually impaired by an increased amount of HIS-HAT1 (Fig 3F). Likewise, HIS-HAT1 also weakened the interaction between HIS-MYB75 and MBP-EGL3 (Fig 3F). Using protoplasts from *35S*:*HAT1 #13*, we found that the interaction between TT8/EGL3 and MYB75 is counteracted by endogenous HAT1-GFP (Fig 3G). We further tested whether HAT1 interferes with the interaction between MYB75 and TT8 or EGL3 in a yeast three-hybrid assay. When yeast transformant that carried both AD-MYB75 and pBridge-TT8-HAT1 plasmids were grown on plates with high methionine concentrations (200 μM), which restrain the expression of HAT1, MYB75 strongly interacted with TT8 (Fig 3H). When the level of methionine was reduced from 200 μM to 50 μM, hence permitting HAT1 expression, yeast growth were consumingly suppressed in these transformants (Fig 3H). Similar results were observed when yeast transformed with AD-MYB75 and pBridge-EGL3-HAT1 (Fig 3H). In summary, these results prove that HAT1 interferes with the formation of MBW protein complex.

**Fig 4:**
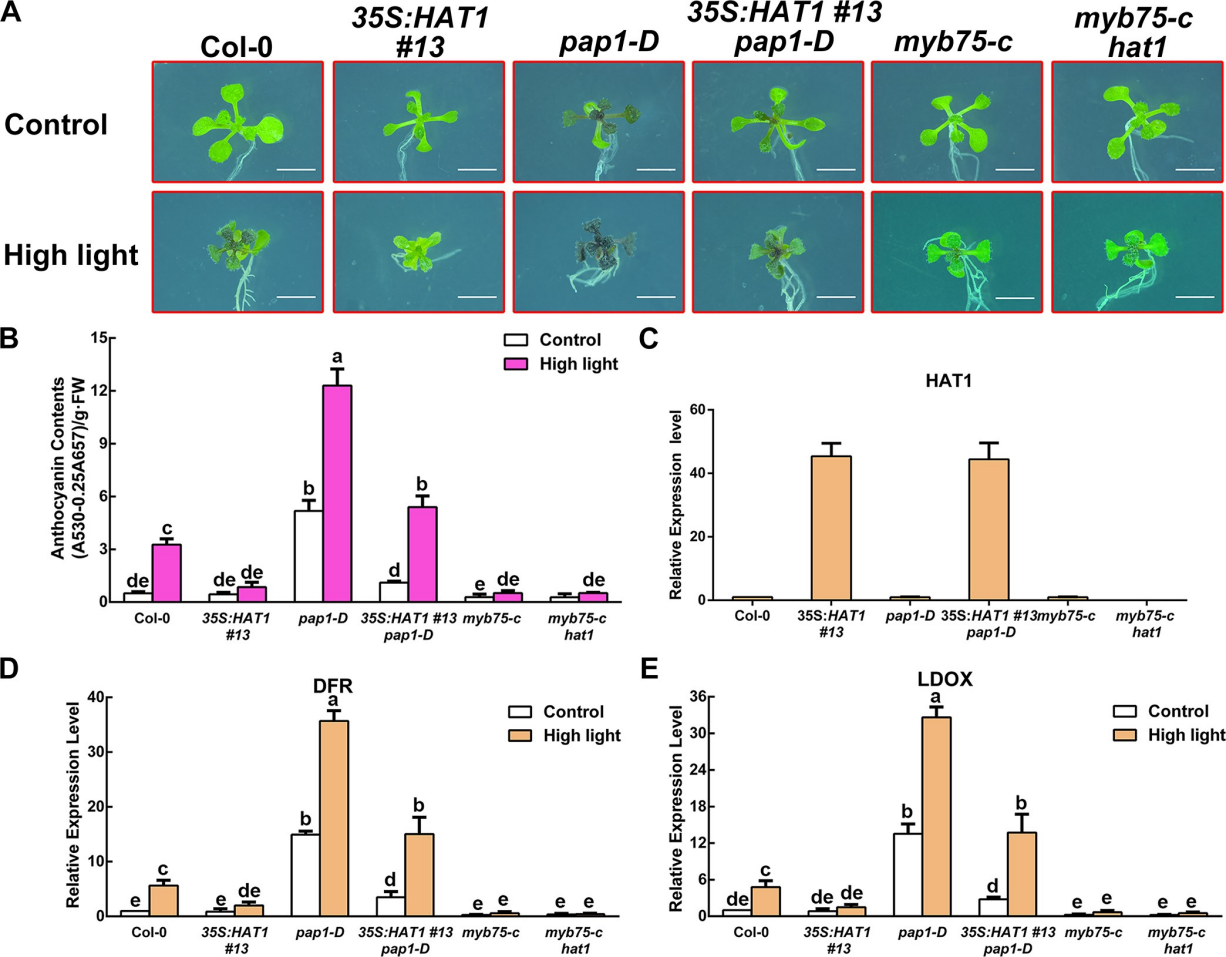
Overexpression of HAT1 represses anthocyanin accumulation in *pap1-D* mutant. (A) 14-day-old Arabidopsis seedlings of Col-0, *35S*:*HAT1 #13*, *pap1-D*, *35S*:*HAT1 #13 pap1-D*, *myb75-c*, *myb75-c hat1* grown on plates under different conditions. Bars = 0.5 cm. (B) Anthocyanin levels in extracts from seedlings in (A). The experiments were performed in biological triplicate (representing anthocyanin content measured from 15 plants of each genotype and treatment were pooled for one replicate). FW, fresh weight. Error bars denote ± SD (n = 3). Different letters represented statistically significant differences (two-way ANOVA, p<0.05). (C) qPCR analysis of HAT1 transcript levels in 14-day-old seedlings in (A) grown on plates under control conditions, respectively. Expression levels were standardized to *ACTIN 8*, and results in the Col-0 under control conditions were set at 1. Error bars denote ± SD (n = 3). (D-E) qPCR analysis of *DFR*, *LDOX*, and *UF3GT* expression levels in 14-day-old seedlings in (A) grown on plates under different conditions for 9 h, respectively. Expression levels were standardized to *ACTIN 8*, the results of Col-0 under control conditions were set at 1. Error bars denote ± SD (n = 3). Different letters represented statistically significant differences (two-way ANOVA, p<0.05).

Next the transgenic line *pap1-D* overexpressing *MYB75* was crossed with *35S*:*HAT1#13* to generate the *pap1-D 35S*:*HAT1 #13* plants. As expected, overexpression HAT1 repressed anthocyanin accumulation in *pap1-D* background under control or high light conditions (Fig 4A and 4B). We also crossed *hat1* null mutant with *myb75-c* that *MYB75* knockout mutant using the CRISPR-Cas9 system. Under high light conditions, the *myb75-c hat1* double mutant exhibited similar phenotype with *myb75-c*. Consistent with the phenotype, transcript levels of anthocyanin biosynthetic genes *DFR* and *LDOX* were also lower in *pap1-D 35S*:*HAT1 #13* than that of *pap1-D* mutant under normal or high light conditions, although the expression levels of HAT1 in *pap1-D 35S*:*HAT1 #13* were similar with *pap1-D* (Fig 4C–4E). Additionally, the anthocyanin content of the *pap1-D hat1* exhibited no significant difference than that of *pap1-D* under high light conditions (S5A and S5B Fig), it might be due to high light repressed HAT1 expression in *pap1-D* plants (Fig 1D). Meanwhile, *35S*:*HAT1 #13* and *35S*:*HAT1 #13 myb75-c* showed a similar anthocyanin accumulation phenotype (S5C and S5B Fig). Taken together, our results suggest that HAT1 represses anthocyanin accumulation through interacting with MYB75 and at least partially by interfering with the formation of MBW protein complex.

### HAT1 interacts with TPL and behaves as a transcriptional repressor

**Fig 5:**
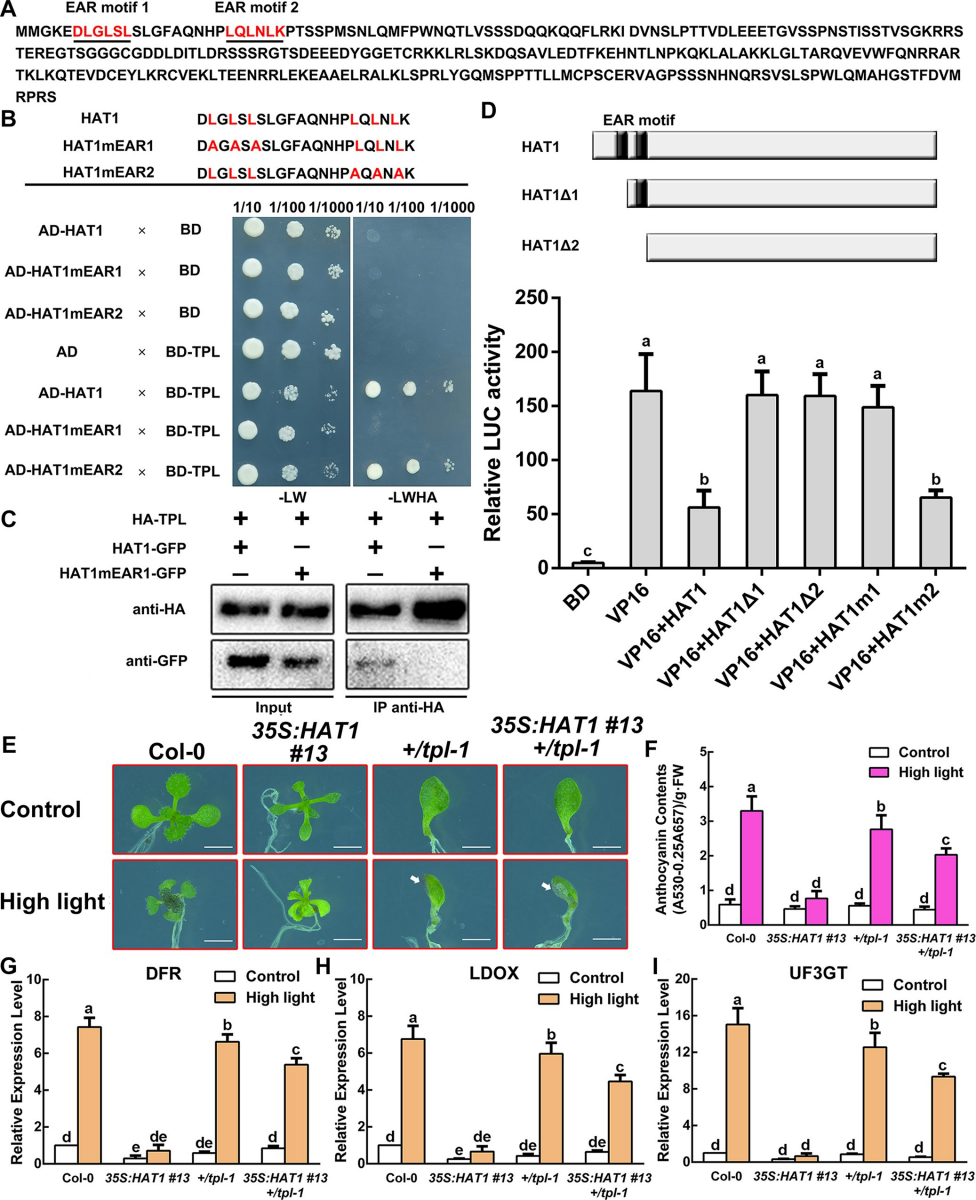
HAT1 interacts with TPL and behaves as a transcriptional repressor. (A) The amino acid sequence of HAT1 (*At4g17460*). HAT1 contains two possible EAR motif (underlined, red). (B) Interaction between HAT1 and TPL protein in yeast two-hybrid assays. HAT1mEAR, mutated HAT1 in which the core Leu residues of the EAR motif were substituted to Ala residues. The ability of cells to grow on synthetic dropout medium lacking Leu, Trp, His, and Ade (-LWHA) suggested the interaction. BD, GAL4 DNA binding domain. (C) CoIP analysis for HAT1 and TPL. Co-IP was performed using transgenic Arabidopsis plants co-expressing 35S:HAT1-GFP and 35S:HA-TPL or co-expressing 35S:HAT1mEAR1-GFP and 35S:HA-TPL. HAT1-GFP and HA-MYB75 were detected with the anti-GFP and anti-HA antibodies, respectively. (D) Transcriptional inhibition assays of HAT1 in protoplast. HAT1Δ1 or HAT1Δ2, the EAR motif was deleted in HAT1. HAT1m1 or HAT1m2, mutated HAT1 that the core Leu residues were substituted to Ala residues in EAR motif. BD, empty vector, negative control. VP16, a herpes simplex virus-encoded transcriptional activator protein, positive control. ns, no significant difference; Error bars denote ± SD (n = 3). Different letters represented statistically significant differences (one-way ANOVA, p<0.05). (E) 14-day-old Arabidopsis seedlings of Col-0, 35S:HAT1 #13, +/tpl-1, 35S:HAT1 #13 +/tpl-1 grown on plates under different conditions. Arrowheads represent anthocyanin accumulation in +/tpl-1, 35S:HAT1 #13 +/tpl-1 seedlings. Bars = 0.5 cm. (F) Anthocyanin levels in extracts from seedlings in (E). The experiments were performed in biological triplicate (representing anthocyanin content measured from 15 plants of each genotype and treatment were pooled for one replicate). FW, fresh weight. Error bars denote ± SD (n = 3). Different letters represented statistically significant differences (two-way ANOVA, p<0.05). (G-I) qPCR analysis of DFR, LDOX, and UF3GT expression levels in 14-day-old seedlings in (E) grown on plates under different conditions for 9 h, respectively. Expression levels were standardized to ACTIN 8, the results of Col-0 under control conditions were set at 1. Error bars denote ± SD (n = 3). Different letters represented statistically significant differences (two-way ANOVA, p<0.05).

Although we have proved that HAT1 represses anthocyanin accumulation by sequestering the formation of MBW protein complex, we hypothesize that MYB75-HAT1 complex behaves as a repressor because overexpressing HAT1 in *pap1-D* represses anthocyanin accumulation when compared with *pap1-D* mutant. The previous study demonstrated that the members of HD-Zip II family act as a repressor in modulation of gene expression [31]. HAT1 protein possesses a leucine zipper motif (LZ, between amino acids residue 190 and 233) followed by a DNA-binding homeodomain (HD, between amino acids residue 134 and 188) (Fig 2B). Interestingly, two typical ERF-associated-amphiphilic repression (EAR) motif (DLGLSL and LQLNLK), which is proved as repression motif to repress transcription [49], are located at the N-terminal end of the HAT1 protein (Fig 5A). Many transcriptional repressors have been proved to regulate plant developmental process and signaling pathways by interacting with corepressor TPL/TRRs via EAR motif [40, 41, 43]. This promoted us to investigate whether HAT1 interacted with TPL/TRRs. Firstly, yeast two-hybrid assay showed that HAT1 interacted with TPL (Fig 5B). Furthermore, the interaction relied on the HAT1 EAR motif 1, because replacement of the three conserved Leu residues into Ala residues (LxLxL to AxAxA, designated as HAT1mEAR) abolished the interaction (Fig 5B). We also found that HAT1 interacted with TPR3, but not with TPR1, TPR2 and TPR4 (S6 Fig). Next we used Co-IP experiments to further check the interaction. Consistent with the yeast two-hybrid assays, Co-IP assays proffered that HAT1 interacts with TPL in plants, but HAT1mEAR1 could not form a complex with TPL (Fig 5C), suggesting that EAR motif 1 determined the interaction between HAT1 and TPL. Finally, we investigated whether HAT1 behaved as a transcriptional repressor to inhibit VP16-mediated transcriptional activation. As expected, wild-type HAT1 significantly inhibit the VP16-promoted LUC activity, but deletion of the EAR motif 1 (HAT1ΔEAR) or HAT1mEAR1 abolished the inhibitory effects (Fig 5D). Taken together, these results suggested that HAT1 interacts with TPL and plays as a repressor.

We next investigated whether the HAT1-dependent repression of anthocyanin accumulation is released by the loss of function of TPL. To clarify this, we crossed *35S*:*HAT1 #13* to the *tpl-1* mutant, which is an N176H substitution and has a dominant-negative on the rest of TPRs [50]. Loss of function of TPL in *35S*:*HAT1 #13* (*35S*:*HAT1 #13 +/tpl-1*) largely rescued the lower anthocyanin accumulation phenotype of *35S*:*HAT1 #13* under high light conditions (Fig 5E and 5F). Consistently, repression of *DFR*, *LDOX* and *UF3GT* in *35S*:*HAT1 +/tpl-1* were largely released than that of *35S*:*HAT1 #13* (Fig 5G–5I). These data suggest that HAT1 represses anthocyanin accumulation partially by interacting with TPL/TPRs.

**Fig 6:**
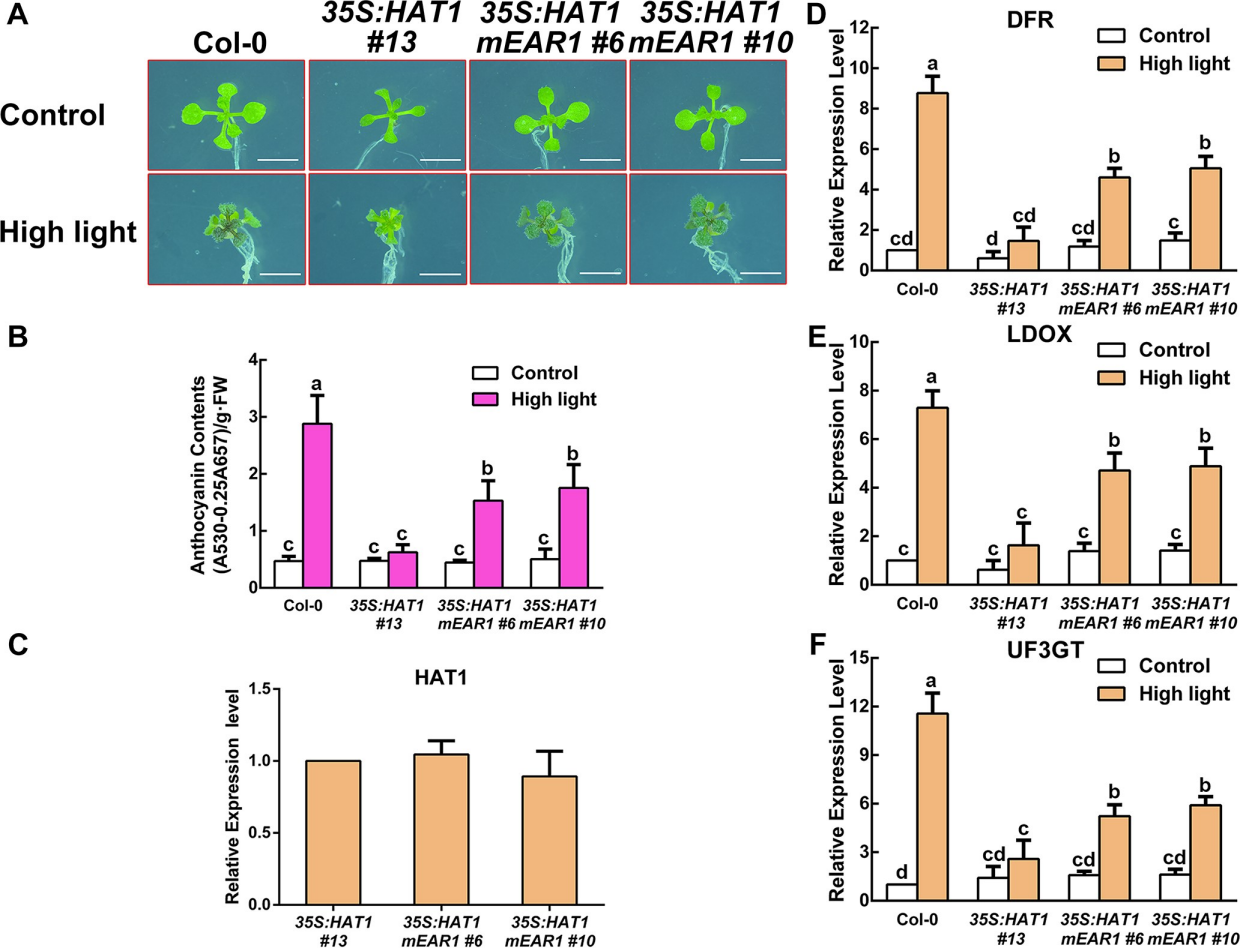
The EAR motif 1 of HAT1 is necessary for repression of anthocyanin accumulation. (A) 14-day-old Arabidopsis seedlings of Col-0, *35S*:*HAT1 #13*, *35S*:*HAT1mEAR1 #6*, *35S*:*HAT1mEAR1 #10* grown on plates under different conditions. Bars = 0.5 cm. (B) Anthocyanin levels in extracts from seedlings in (A). The experiments were performed in biological triplicate (representing anthocyanin content measured from 15 plants of each genotype and treatment were pooled for one replicate). FW, fresh weight. Error bars denote ± SD (n = 3). Different letters represented statistically significant differences (two-way ANOVA, p<0.05). (C) qPCR analysis of HAT1 transcript levels in 14-day-old seedlings in (A) grown on plates under control conditions, respectively. Expression levels were standardized to *ACTIN 8*, and results in the Col-0 under control conditions were set at 1. Error bars denote ± SD (n = 3). (D-F) qPCR analysis of *DFR*, *LDOX*, and *UF3GT* expression levels in 14-day-old seedlings in (A) grown on plates under different conditions for 9 h, respectively. Expression levels were standardized to *ACTIN 8*, the results of Col-0 under control conditions were set at 1. Error bars denote ± SD (n = 3). Different letters represented statistically significant differences (two-way ANOVA, p<0.05).

To further illustrate the mechanism how HAT1 regulated anthocyanin accumulation, we generated transgenic plants overexpressing variants of HAT1 protein (*35S*:*HAT1mEAR1*). This approach was used successfully in the previous study [43]. Two independent transgenic lines, *35S*:*HAT1mEAR1 #6* and *35S*:*HAT1mEAR1 #10*, showed more anthocyanin accumulation than of *35S*:*HAT1 #13* under high light conditions (Fig 6A and 6B). Intriguingly, we noticed that the anthocyanin contents of *35S*:*HAT1mEAR1 #6* and *35S*:*HAT1mEAR1 #10* could not completely restore to that of wild-type plants. Consistently, the expression levels of these three transgenes were similar, but the transcript levels of *DFR*, *LDOX* and *UF3GT* were higher in the *35S*:*HAT1mEAR1* transgenic plants under high light conditions compared with *35S*:*HAT1 #13* plants (Fig 6C–6F). These data indicate the EAR motif 1 is required for HAT1-repressed anthocyanin accumulation.

**Fig 7:**
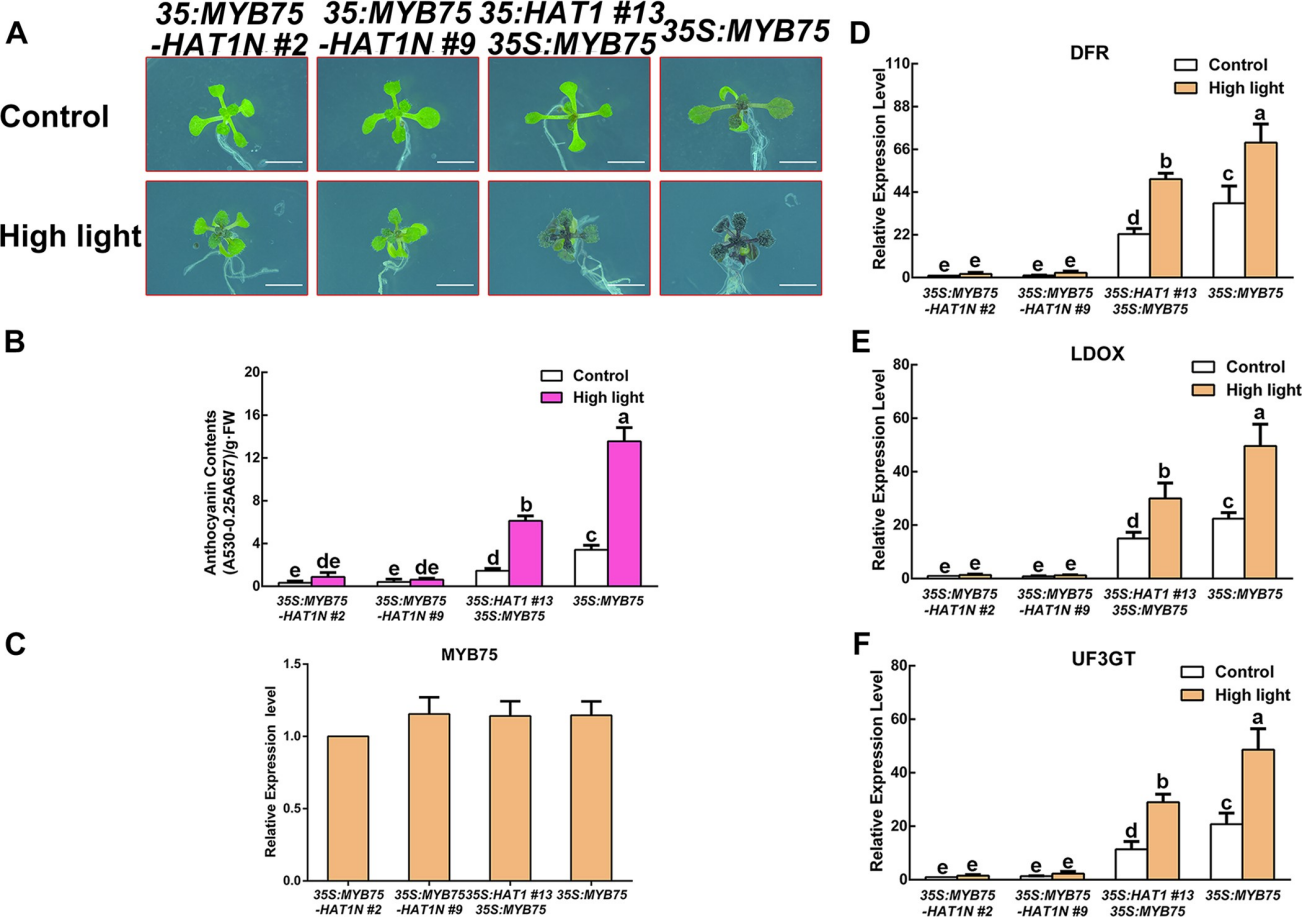
Plants overexpressing MYB75-HAT1N fusion protein exhibit an opposite phenotype contrast to plants overexpressing MYB75. (A) 14-day-old Arabidopsis seedlings of *35S*:*MYB75-HAT1N #2*, *35S*:*MYB75-HAT1N #9*, *35S*:*HAT1 #13 35S*:*MYB75*, *35S*:*MYB75* grown on plates under different conditions. Bars = 0.5 cm. (B) Anthocyanin levels in extracts from seedlings in (A). The experiments were performed in biological triplicate (representing anthocyanin content measured from 15 plants of each genotype and treatment were pooled for one replicate). FW, fresh weight. Error bars denote ± SD (n = 3). Different letters represented statistically significant differences (two-way ANOVA, p<0.05). (C) qPCR analysis of MYB75 transcript levels in 14-day-old seedlings in (A) grown on plates under control conditions, respectively. Expression levels were standardized to *ACTIN 8*, and results in the Col-0 under control conditions were set at 1. Error bars denote ± SD (n = 3). (D-F) qPCR analysis of *DFR*, *LDOX*, and *UF3GT* expression levels in 14-day-old seedlings in (A) grown on plates under different conditions for 9 h, respectively. Expression levels were standardized to *ACTIN 8*, the results of Col-0 under control conditions were set at 1. Error bars denote ± SD (n = 3). Different letters represented statistically significant differences (two-way ANOVA, p<0.05).

It has been reported that transgenic plants overexpressing MYB75-SRDX fusion protein (35S:MYB75-SRDX) exhibited minimal anthocyanin under 3% sucrose [51]. Like EAR motif, the SRDX domain can convert transcriptional activators into dominant repressor when fused to transcription factors. In our study, the evidence that the C-terminal fragment of HAT1 (234 to 282 residues) interacts with MYB75 and that the EAR motif in the N-terminal region interacts TPL suggests that HAT1 could link TPL to MYB75 and convert MYB75 into a repressor. To check this hypothesis, MYB75 was fused with the N-terminal fragment of HAT1 (1 to 90 residues containing EAR motif), and the fusion construct driven by a CaMV 35S promoter was transformed into wild-type Arabidopsis (designated *35S*:*MYB75-HAT1N*). As expected, two independent transgenic plants, *35S*:*MYB75-HAT1N #2* and *35S*:*MYB75-HAT1N #9*, exhibited minimal anthocyanin accumulation compared with *35S*:*MYB75* transgenic plants under high light conditions (Fig 7A and 7B). Consistently, the levels of *DFR*, *LDOX* and *UF3GT* transcripts were also lower in *35S*:*MYB75-HAT1N #2* and *35S*:*MYB75-HAT1N #9* plants compared with *35S*:*MYB75* transgenic plants under high light conditions despite the similar expression levels of MYB75 in these transgenes (Fig 7C–7F).

Then we performed a transient transformation assay using the *DFR* promoter fused to the LUC gene as a reporter. HA-MYB75, HA-TPL and HAT1-GFP construct acted as effector and transfected together with the reporter construct into *myb75-c* mesophyll protoplasts. The LUC expression of DFR promoter was very low without MYB75 expression, but was activated by expression of MYB75 (S7 Fig). However, when HAT1 was coexpressed with MYB75, this activation was markedly decreased (S7 Fig). The relative luciferase activities were moreover decreased when TPL was co-transformed with HAT1, but the repression was alleviated when TPL was coexpressed with mutational HAT1, suggesting that HAT1 inhibits the transcriptional activity of MYB75 through TPL function. Taken together, these results indicate that HAT1 represses anthocyanin accumulation by connecting TPL with MYB75.

### HAT1 acts as a repressor by impairing target genes histone H3 acetylation

**Fig 8:**
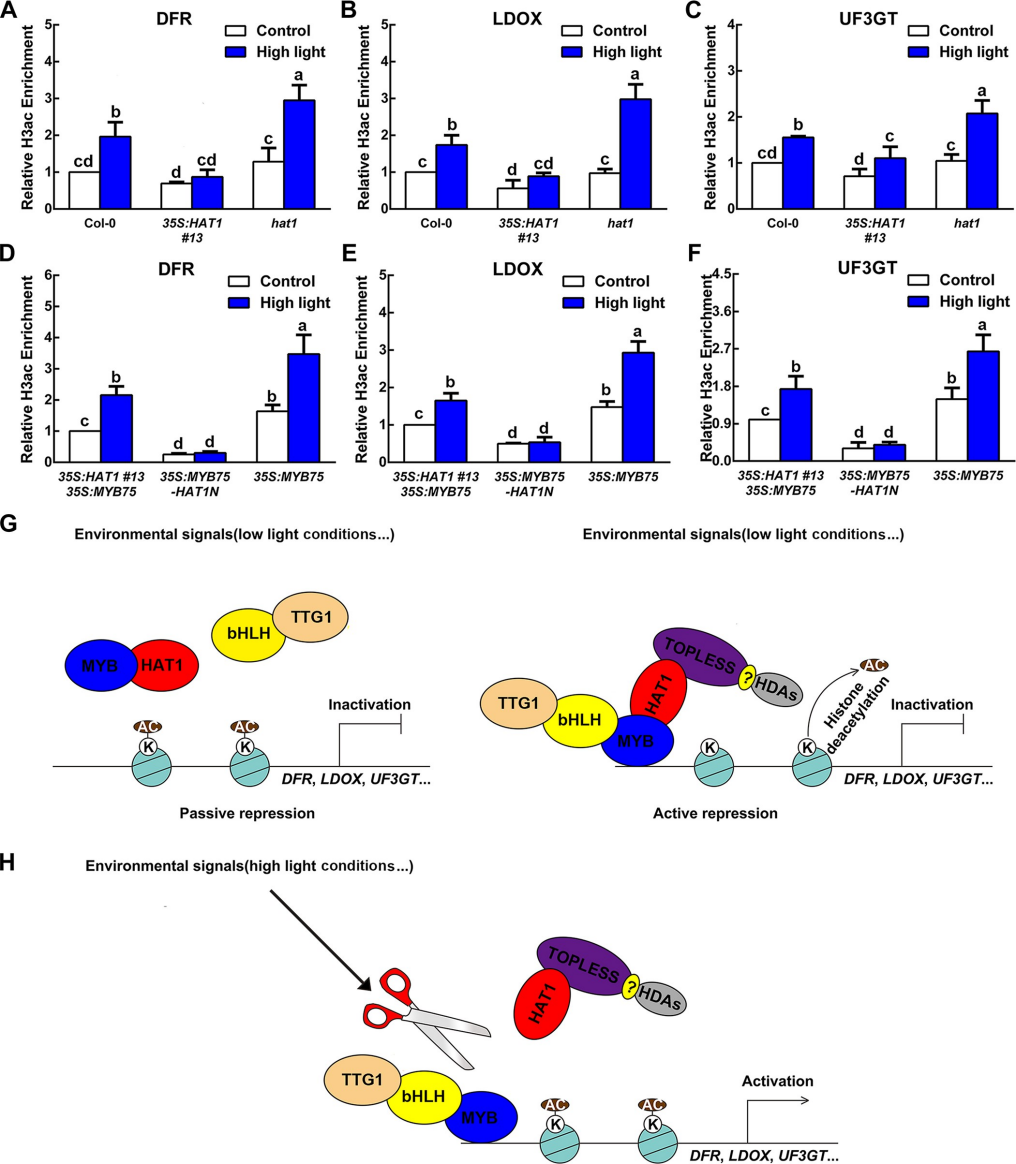
Overexpression of HAT1 impairs histone H3 acetylation under high light conditions and a working model for HAT1 in repressing anthocyanin accumulation. (A-C) ChIP-qPCR determines the histone H3 acetylation levels in the transcription start sites (TSSs) of *DFR*, *LDOX*, and *UF3GT* in Col-0, *35S*:*HAT1 #13*, and *hat1* under different conditions. The histone H3 acetylation levels were standardized to *ACTIN 7* and results in the Col-0 under control conditions were set at 1. Error bars denote ± SD (n = 3). Different letters represented statistically significant differences (two-way ANOVA, p<0.05). (D-F) ChIP-qPCR determines the histone H3 acetylation levels in the transcription start sites (TSSs) of *DFR*, *LDOX*, and *UF3GT* in *35S*:*MYB75-HAT1N #2*, *35S*:*HAT1 #13 35S*:*MYB75*, and *35S*:*MYB75* under different conditions. The histone H3 acetylation levels were standardized to *ACTIN 7* and results in the *35S*:*HAT1 #13 35S*:*MYB75* under control conditions were set at 1. Error bars denote ± SD (n = 3). Different letters represented statistically significant differences (two-way ANOVA, p<0.05). (G) Under non-inductive conditions, such as low light conditions, plants possess high levels of HAT1. Meanwhile, HAT1 interferes with MBW protein complex by binding MYB75 (Left), as well as transforming the active MBW protein complex into a repressive complex by recruiting EAR motif-dependent TPL corepressor (Right), thus preventing the expression of LBGs. (H) Under inductive conditions, such as high light conditions, HAT1 expression is suppressed. MYB75 and bHLH transcription factors are able to form MBW protein complex with TTG1 protein, and finally activates late biosynthetic genes expression.

Previous studies have shown that TPL interacts with two histone deacetylase, HDA6 and HDA19, which function in chromatin modification and epigenetic regulation of developmental and hormone-responsive genes [52, 53]. The presence of ternary MYB75-HAT1-TPL protein complex led us to further analyze whether HAT1 represses the expression of anthocyanin biosynthetic genes via chromatin modifications. To prove this speculation, ChIP assays were performed by using antibody of acetylated H3 in different mutants. As shown in Fig 8A–8C, reduced histone H3 acetylation was verified in the transcription start sites (TSSs) of *DFR*, *LDOX* and *UF3GT* in *35S*:*HAT1 #13* under high light conditions, while elevated histone H3 acetylation was detected in *hat1* mutant under the same condition. Simultaneously, *35S*:*MYB75-HAT1N #2* transgenic plants showed lower histone H3 acetylation in the TSSs of *DFR*, *LDOX* and *UF3GT* than that of *35S*:*MYB75* transgenic plants (Fig 8D–8F). These fingdings suggest that MYB75-HAT1-TPL inhibits LBGs expression through recruiting a histone modification complex.

## Discussion

Anthocyanins ara one kind of flavonoids induced by environmental stimuli and developmental signals. Exposed to extreme conditions, such as high light, high concentration of sucrose, and drought, plants evolved a range of mechanisms to regulate transcription of anthocyanin biosynthetic genes [29]. The spatial and temporal regulation of anthocyanin accumulation are determined by an MBW protein complex, while the R2R3-MYBs are pivotal to appoint the function of the MBW protein complex [14]. Here we illustrated that HAT1 negatively regulated anthocyanin accumulation through hindering the active function of MBW protein complex and epigenetic regulation.

### HAT1 negatively regulates anthocyanin accumulation in Arabidopsis

Although members of HD-Zip family have been well described in plants, the possible function in anthocyanin accumulation remains to investigate. GL2, a member of HD-Zip subfamily IV, has been characterised as a negative regulator of anthocyanin accumulation in Arabidopsis [54]. In this study, our findings elucidate that HAT1 may function as a new repressor in anthocyanin accumulation through sequestering the MBW protein complex and epigenetic regulation in Arabidopsis. Additionally, *MYB75*, *MYB90*, *TT8* and *EGL3* transcript levels were increased in *hat1* mutant and decreased in *35S*:*HAT1* plants (S2D Fig), presenting the possibility that HAT1 regulates anthocyanin accumulation by modulating MBW protein complex expression. Our results indicated that HAT1 regulated anthocyanin accumulation via post-translational regulation and transcriptional regulation of MBW protein complex.

Much work so far has evidenced that the expression of the members of HD-Zip II family is induced in response to simulated shade conditions. Interestingly, weak anthocyanin accumulation occurs under shade conditions in petunia plants and the conversion of light conditions change the members of HD-Zip II family abundance [30, 55]. It seems that the members of HD-Zip II family are involved in modulation of anthocyanin accumulation in response to light/shade. However, MYB75 did not interact with HAT2, HAT3, ATHB2, and ATHB4 in yeast (S8 Fig), suggesting that HAT1 is a unique gene of HD-Zip II family to repress anthocyanin accumulation.

### HAT1 is a transcriptional repressor involved in hormone signaling and developmental process

It is likely that overexpression of one member will repress the expression of rest in HD-Zip family II [34]. Previous study described that the EAR motif exists in different members of HD-Zip II family [30]. We investigate the impact of EAR motif on anthocyanin accumulation. We proved that EAR motif is required for HAT1-repressed anthocyanin accumulation. Further, HAT1 suppresses anthocyanin accumulation via TPL-dependent histone deacetylation (Fig 8A–8F). Interestingly, our previous study proved that HAT1 negatively regulates hormone synthesis and response gene [36, 45].

It seems that HAT1 behaves as a transcriptional repressor in various hormonal signaling and metabolic process. Our recent study reported that phosphorylation of HAT1 by SnRK2.3 induced its degradation. And abscisic acid (ABA) positively regulated anthocyanin accumulation [56]. It raised the possibility that ABA induced anthocyanin accumulation by inhihiting HAT1 founction. Our previous studies also revealed that HAT1 was a positive regulator in BR pathway [36]. Under favorable conditions, HAT1 accumulated and interacted with BES1 to promote plant growth. Meanwhile, HAT1 also interacted with MYB75 and TPL to inhibit anthocyanin accumulation. It demonstrated that HAT1 was a key regulator in balancing plant growth and stresses adaption.

Numerous EAR motif-contained transcription factors repress gene expression through directly binding to the *cis* element. The previous study showed HAT1 interacts with BES1 [36]. Intriguingly, BES1-TPL complex mediates the inhibitory action of brassinosteroids on ABA responses during early seedling development [57]. We speculate that the these proteins can form a HAT1-BES1-TPL protein complex to repress BR-response gene. A recent study revealed that ATHB4 regulates leaf polarity and hypocotyl elongation by interacting with TPL protein [58]. Several studies proposed that some transcription factors repressed gene transcription by recruiting TPL and HDAs to the promoter of target gene [44, 53, 59]. It is important to highlight that HAT1 inhibits the expression of anthocyanin biosynthetic-specific genes through bridging MYB75 and TPL rather than directly binding to the promoter of these genes.

### HAT1 represses anthocyanin accumulation in stem-rosette junction and leaves during plant senescence

Anthocyanin accumulation occurs at the junction of the rosette leaves and stem during plant development in Arabidopsis [28]. We noticed the purple pigments of *35S*:*HAT1 #13* plants was much lower than that of wild-type, whereas *hat1* mutant exhibited more purple pigments compared with wild-type (S9A and S9B Fig). The expression of *DFR* is confined within basal regions of stems during the transition from leaves to flowers [28]. MYB75 showed high expression in the lower part of the inflorescence stem and leaves in 6-week-old Arabidopsis [48]. Our results showed that transcript levels of HAT1 was minimal in the lower part of the inflorescence stem and highest in the upper part of the inflorescence stem, while DFR and MYB75 exhibited an inverse pattern in 6-week-old Arabidopsis (S10A Fig). Consequently, low expression of HAT1 eliminates the repression for MBW protein complex, thus resulting in anthocyanin accumulation in stem-rosette junction. On the other hand, HAT1 showed low expression in senescent leaves, while DFR and MYB75 displayed high expression in senescent leaves (S10B Fig). Consistent with this, gene expression analysis using the Arabidopsis microarray data displayed in the eFP browser indicated opposite expression patterns of MYB75 and HAT1, suggesting HAT1 serves as a repressor in senescent leaves during plant senescence (S11 Fig). MBW protein complex activates anthocyanin biosynthetic-specific gene expression when HAT1 expression is limited and thereby induces anthocyanin accumulation. These data proved that HAT1 represses anthocyanin accumulation in stem-rosette junction and leaves during plant senescence.

### Epigenetic modifications fine-tune the anthocyanin accumulation

Present studies demonstrated that epigenetic modifications participated in regulation of anthocyanin accumulation [60, 61]. Recent research suggested DELLA promoted anthocyanin accumulation via sequestering MYBL2 and JAZ suppressors of the MBW complex in Arabidopsis thaliana [62]. To test the relationship between HAT1 and MYBL2 in suppression of anthocyanin accumulation, we crossed *35S*:*HAT1* #13 with *mybl2* mutant. The *35S*:*HAT1 #13 mybl2* plants showed less anthocyanin accumulation (S12A and S12B Fig), indicating that HAT1 repressed anthocyanin accumulation is independent of the MYBL2-regulated pathway. We further observed that H3 acetylation levels in the promoter of LBGs were elevated in *mybl2* mutant under high light conditions (S12C–S12E Fig). These results suggest that epigenetic modifications are prevalent in regulation of anthocyanin accumulation in plants. Interestingly, maize bHLH transcription factor *R* interacts with *RIF1* and thereby forms a complex with MYB transcription factor *C1*. C1-R-RIF1 complex binds to A1 promoter and activates A1 expression by elevating the H3K9 and H3K14 acetylation levels in the promoter region [63]. Therefore, it remains challenging to identify the interaction between MBW protein complex and epigenetic regulators, which may explain why MBW protein complex activates the expression of biosynthetic genes.

### A working model for HAT1 in repressing anthocyanin accumulation

To date, much work has characterized many repressors in modulation of anthocyanin accumulation. Several repressors regulate anthocyanin accumulation without direct interaction with members of MBW protein complex [7, 54, 64]. Notably, it has been proved that LBD37 interact with TPL via Y2H screening [65]. On the other side, MYB75, bHLH and TTG1 are expressed in various tissues of Arabidopsis under non-inductive conditions [15, 16, 19, 48]. Therefore, some repressors suppress anthocyanin accumulation by directly interacting with members of MBW protein complex [4, 28, 29, 66]. We prove that HAT1 suppresses anthocyanin accumulation by directly interacting with MYB75. Intriguingly, the proteins that interact with MBW protein complex all contain EAR moif except SPL9. Y2H screening proved that JAZ5/JAZ6/JAZ7/JAZ8, MYBL2 and HAT1 interact with TPL respectively [65]. We speculate that EAR moif containd-repressors inhibit anthocyanin accumulation by forming a protein complex with components of MBW and TPL in plants. The repressive activity of MYBL2 might play a critical role in suppression of anthocyanin accumulation [24]. In our studuy, HAT1mEAR1 may still interact with MYB75 and interfere with MBW protein complex because the C terminus of HAT1 determines the interaction with MYB75. We observe that anthocyanin level of *35S*:*HAT1mEAR1* transgenic plants was lower than that of wild-type. These results suggested that HAT1 inhibits anthocyanin accumulation partially by disturbing the formation of MBW protein complex. We suppose that interference of MBW protein complex (Passive repression) and epigenetic modification (Active repression) function synchronously in regulation of anthocyanin accumulation.

Under non-inductive conditions, such as low light conditions, plants possess high levels of HAT1. Meanwhile, HAT1 interferes with MBW protein complex by binding MYB75, as well as transforming the active MBW protein complex into a repressive complex by recruiting EAR motif-dependent TPL corepressor, thus preventing the expression of LBGs (Fig 8G). Under inductive conditions, such as high light conditions, HAT1 expression is suppressed. MYB75 and bHLH transcription factors are able to form MBW protein complex with TTG1 protein, which activates transcription of the target genes encoding LBGs (Fig 8H). Overall, our work together with other studies suggests that plants restrict the expression of anthocyanin biosynthetic genes via the multiple and intricate mechanisms.

## Methods

### Plant materials and growth conditions

The Arabidopsis thaliana *35S*:*HAT1 #11*, *35S*:*HAT1 #13* and *hat1* mutants were described as previously [36]. All wild-type, various mutants, and transgenic plants in this study are in Col-0 ecotype background. To avoid ecotype variability, the *tpl-1* mutant, originally in Ler background [40], was introgressed into the Col-0. Arabidopsis seeds were placed on half-strength Murashige and Skoog medium. The plates were placed at 4°C for 3 d avant transfering to 22°C under different light conditions. Plates were put at control (40 μmol m^-2^ s^-1^) or high light (180 μmol m^-2^ s^-1^) conditions with a 16-h-light/8-h-dark photoperiod for high light-induced research [5]. For the BiFC assays, *Nicotiana benthamiana* was cultured in soil at 22°C under 16-h-light/8-h-dark conditions.

### Anthocyanin measurement

Anthocyanin levels were measured as described previously [67]. Briefly, arabidopsis seedlings were incubated in extraction buffer (methanol containing 1% HCl) overnight at 4°C in the dark. After extraction and centrifuged, the supernatants were collected and absorbance calculated at 530 and 657 nm. Relative anthocyanin content was quantified by (A_530_-0.25×A_657_) per gram fresh weight.

### Plasmid construction and plant transformation

HAT1mEAR1 was amplified from pZP211-HAT1 using primers indicated in S1 Table and cloned into pZP211 vector to generate 35S:HAT1mEAR1-GFP [68]. To generate MYB75 constructs, the 1500 bp genomic sequence of MYB75 contained the coding area was cloned into pCM1307 vector to create 35S:HA-MYB75 [69]. The sequence coding N terminus of HAT1 was amplified from pZP211-HAT1 and cloned into pCM1307-MYB75 to generate 35S:HA-MYB75-HAT1N. The coding sequences (CDS) of TPL were amplified and cloned into pCM1307 plasmid to create 35S:HA-TPL. Oligo primers used for cloning are listed in S1 Table. Col-0 plants were transformed with these constructs by using Agrobacterium tumefaciens (strain GV3101)-mediated transformation [70].

### Transcriptional inhibition assays in protoplast

The GAL4 reporter plasmid was generated from pUC19 [71], which contains the firefly LUC reporter gene driven by the minimal TATA box of the 35S promoter plus five GAL4 binding elements. For transcriptional inhibition assays, HAT1ΔEAR and HAT1mEAR were amplified from pZP211-HAT1 and cloned into pRT-BD to generate GAL4-HAT1ΔEAR and HAT1mEAR respectively [72]. The positive control (pRT-35S-BD-VP16) was constructed by insertion of VP16, a herpes simplex virus-encoded transcriptional activator protein, into pRT-BD. Plasmid pTRL was used as internal control. For transcription activity assays in protoplast, a 512-bp DFR promoter was amplified from genomic DNA and fused with pGreenII 0800-LUC. The internal control, effectors and reporter were co-transformed into Arabidopsis protoplasts by PEG/CaCl_2_-mediated transfection [73]. All transfection cultured for 16 h, then luciferase assays were performed using the Promega dual-luciferase reporter assay system and a GloMax 20–20 luminometer (Promega, http://www.promega.com). Relative LUC activity was defined as firefly LUC activity divided by Renilla LUC activity.

### Yeast two-hybrid and three-hybrid assays

For yeast two-hybrid assays, the full-length CDS of MYB75 and HAT1 were amplified and cloned into pGADT7 (Clontech). The full-length CDS of MYB75 and N terminus of TPL were amplified and cloned into pGBKT7 (Clontech). The yeast strain (AH109) was transformed with pairs of plasmids and grown on Double DO supplement (SD-Leu/-Trp) for 3 days, then the cotransformants were shifted onto Quadruple DO supplement (SD-Leu/-Trp/-Ade/-His) to test for possible interactions.

For yeast three-hybrid assays, the complete CDS of TT8 and EGL3 were amplified and fused with pBridge-HAT1 plasmid to generate pBridge-TT8-HAT1 and pBridge-EGL3-HAT1 respectively [74]. Yeast three-hybrid experiments performed as described previously [20]. Briefly, pBridge-TT8-HAT1 or pBridge-EGL3-HAT1 were used in co-transformation with pGADT7-MYB75. pBridge contains a methionine (Met) suppressible promoter positioned upstream of a Gateway cassette. HAT1 expression was gradually suppressed using increasing methionine concentrations in Minimal Media Quadruple Dropouts (SD-Leu/-Trp/-His/-Met). For each combination, 3 colonies selected on dropout medium (SD -Leu/-Trp) were resuspended in water, the OD600nm was adjusted to 0.7 and 20 μl was streaked out on the respective plates.

### Bimolecular fluorescence complementation (BiFC) assays

For BiFC assays, full-length CDS of MYB75 and HAT1 was cloned into the pXY103-nYFP vector respectively [75]. The full-length CDS of MYB75, TT8, EGL3 and TTG1 were cloned into the pXY104-cYFP vector respectively [75]. The constructs were transformed into *Agrobacterium tumefaciens* strain (GV3101) respectively, and the lower epidermis of *Nicotiana benthamiana* plants were used for injection of different combination. The transfected plants were grown in the green house for at least 36 hours and fluorescent signals were observed by using scanning microsystem (Leica).

### In vitro pull-down assays

Different version of MYB75 were cloned into the pMAL-C2X and pET28a vectors with MBP tag and 6×His tag respectively. TT8 and EGL3 were also cloned into the pMAL-C2X vector respectively. Different version of HAT1 were cloned into the pMALc-B and pET28a vectors with MBP tag and 6×His tag respectively. In vitro pull-down assays performed as described [76]. Ni-NTA beads containing 5 μg HIS-HAT1 proteins incubated with 5 μg MBP-MYB75 by using 500 μl pull-down buffer (150 mM NaCl, 20 mM Tris, 1 mM PMSF, 0.2% Triton X-100, 1% protease inhibitor cocktail [pH 8.0]) at 4°C for 2 h. Equally, MBP-HAT1 was incubated with Ni-NTA beads contained HIS-MYB75. Beads were washed four times with the pull-down buffer and proteins were eluted from beads by boiling in 95°C with 30 μL SDS-PAGE loading buffer then separated by SDS-PAGE and analyzed by the anti-MBP antibies.

Competitive HIS pull-down analysis performed as described previously [77], 5 μg of MBP-TT8 or MBP-EGL3 mixed with 5, 10, or 20 μg HIS-HAT1 were incubated with Ni-NTA beads containing 5 μg HIS-MYB75 by using 500 μl pull-down buffer (150 mM NaCl, 20 mM Tris, 1 mM PMSF, 0.2% Triton X-100, 1% protease inhibitor cocktail [pH 8.0]) at 4°C for 2 h. Beads were washed four times with the pull-down buffer and proteins were eluted from beads by boiling in 95°C with 30 μL SDS-PAGE loading buffer then separated by SDS-PAGE and analyzed by the anti-MBP antibies.

### Semi-*in vivo* pull-down assay

For the competing MBP pull-down assay [78], samples from Col-0 or 35S:HAT1 protoplasts expressed HA-MYB75 were collected in protein extraction buffer containing 150mM NaCl, 50mM Tris-HCl (pH 7.5), 0.1% (v/v) NonidetP-40, 10% (v/v) glycerol, and 1×complete protease inhibitor cocktail (Roche). The lysate was centrifuged at 12,000 g for 5 min at 4°C, and the supernatant was taken for semi-*in vivo* pull-down assay. MBP-TT8 or MBP-EGL3 beads was added to 500 μL of total extracted protein and incubated at 4°C for 3 h. Beads were washed in extraction buffer five times, resuspended in SDS-PAGE loading buffer and analyzed using SDS-PAGE and immunoblotting with anti-HA antibody, anti-GFP and anti-MBP antibody.

### Co-IP assay

Plants expressing different proteins as indicated were extracted with protein extraction buffer containing 150mM NaCl, 50mM Tris-HCl (pH 7.5), 0.1% (v/v) NonidetP-40, 10% (v/v) glycerol, and 1×complete protease inhibitor cocktail (Roche) [78]. After centrifuging at 12,000 g for 5 min at 4°C, and the supernatant was incubated for 3 h with Anti-HA Agarose Affinity Gel antibody at 4°C. Then the beads were washed six times using extraction buffer and then eluted with 50 μL of SDS-PAGE loading buffer for immunoblot analysis using Anti-HA and Anti-GFP antibody.

### ChIP assays

ChiP assays perform as described [75]. The 4-week-old plants collected in 50 mL tubes, and 37 mL 1% formaldehyde solution was used for cross-linked under a vacuum for 20 min. The chromatin was collected and sheared by sonication to reduce the average DNA fragment size to around 500 bps, then the sonicated chromatin complex was immunoprecipitated by specific antibodies anti-acetyl-histone H3 (Catalog no 06–599, Millipore). After reverse cross-linking, the immunoprecipitated DNA fragment was analysed by qPCR with specific primers shown in S1 Table.

### Real-Time PCR analysis

Total RNA extraction, cDNA synthesis and qRT-PCR were performed as described before [79]. PCR analysis was carried out using SYBR Green PCR Master Mix was used as previously described. Three separate experiments were implemented, and technical triplicates of each experiment were implemented. Gene expression normalize to the transcript levels of *ACTIN 8*.

### Statistical analysis

Samples were analyzed in triplicates, and the data are expressed as the mean ± SD unless noted otherwise. Statistical significance was determined using two-way ANOVA (LSD’s multiple-range test) or Student’s t-test. A difference at P<0.05 was considered significant.

### Accession numbers

The Arabidopsis Genome Initiative identifiers for the genes described in this article are as follows: *HAT1* (At4g17460), *HAT2* (At5g47370), *HAT3* (At3g60390), *ATHB2* (At4g16780), *ATHB4* (At2g44910), *MYB75* (At1g56650), *MYB90* (At1g66390), *TT8* (At4g09820), *EGL3* (At1g63650), *TTG1* (At5g24520), *MYBL2* (At1g71030), *TPL* (At1g15750), *TPR1* (At1g80490), *TPR2* (At3g16830), *TPR3* (At5g27030), *TPR4* (At3g15880), *DFR* (At5g42800), *LDOX* (At4g22880), *UF3GT* (At5g54060), *ACTIN 7* (At5g09810) and *ACTIN 8* (At1g49240).

## Supporting information

S1 Fig Anthocyanin accumulation in 3-week-old plants grown in soil. (A) 3-week-old Arabidopsis plants of Col-0, *35S*:*HAT1 #13*, and *hat1* grown in soil under different conditions. Bars = 1 cm.
(B) Anthocyanin levels in extracts from seedlings in (A). The experiments were performed in biological triplicate (representing anthocyanin content measured from 15 plants of each genotype and treatment were pooled for one replicate). FW, fresh weight. Error bars denote ± SD (n = 3). Different letters represented statistically significant differences (two-way ANOVA, p<0.05).
(TIF)

S2 Fig Anthocyanin accumulation in hypocotyls and cotyledons of Col-0, *35S:HAT1 #11*, *35S:HAT1 #13*, and *hat1* plants. (A) 5-day-old Arabidopsis plants of Col-0, *35S*:*HAT1 #11*, *35S*:*HAT1 #13*, and *hat1* grown in 1/2 MS media under control or 6% sucrose conditions. Bars = 1 mm.
(B) Anthocyanin levels in extracts from seedlings in (A). The experiments were performed in biological triplicate (representing anthocyanin content measured from 15 plants of each genotype and treatment were pooled for one replicate). FW, fresh weight. Error bars denote ± SD (n = 3). Different letters represented statistically significant differences (two-way ANOVA, p<0.05).
(C) qPCR analysis of *DFR*, *LDOX* and *UF3GT* expression levels in 3-day-old seedlings grown on 1/2 MS media. Expression levels were standardized to *ACTIN 8*, and results of Col-0 were set at 1. Error bars denote ± SD (n = 3).
(D) qPCR analysis of anthocyanin regulatory genes transcript levels in 3-day-old seedlings grown on 1/2 MS media. Expression levels were standardized to *ACTIN 8*, and results of Col-0 under control conditions were set at 1. Error bars denote ± SD (n = 3).
(TIF)

S3 Fig HAT1 does no interact with MYB90. HAT1 does not interact with MYB90 in yeast. The ability of cells to grow on synthetic dropout medium lacking Leu, Trp, His, and Ade (-LWHA) suggested the interaction. Interaction between HAT1 and MYB75 served as a positive control. AD, GAL4 activation domain. BD, GAL4 DNA binding domain.
(TIF)

S4 Fig HAT1 does not interact with TT8, EGL3, and TTG1 *in vivo*. No signals of YFP fluorescence were observed in tobacco leaves when co-expression of HAT1-nYFP and TT8-cYFP, HAT1-nYFP and EGL3-cYFP, HAT1-nYFP and TTG1-cYFP. Co-expression of HAT1-nYFP and HAT1-cYFP served as transformation control. Bars = 50 μm.
(TIF)

S5 Fig Anthocyanin accumulation in *pap1-D hat1* and *35S:HAT1 #13 myb75-c* plants. (A) 14-day-old Arabidopsis seedlings of Col-0, *pap1-D*, *hat1*, *pap1-D hat1* grown on plates under different conditions. Bars = 0.5 cm.
(B) Anthocyanin levels in extracts from seedlings in (A). The experiments were performed in biological triplicate (representing anthocyanin content measured from 15 plants of each genotype and treatment were pooled for one replicate). FW, fresh weight. Error bars denote ± SD (n = 3). Different letters represented statistically significant differences (two-way ANOVA, p<0.05).
(C) 14-day-old Arabidopsis seedlings of Col-0, *35S*:*HAT1 #13*, *myb75-c*, *35S*:*HAT1 #13 myb75-c* grown on plates under different conditions. Bars = 0.5 cm.
(D) Anthocyanin levels in extracts from seedlings in (C). The experiments were performed in biological triplicate (representing anthocyanin content measured from 15 plants of each genotype and treatment were pooled for one replicate). FW, fresh weight. Error bars denote ± SD (n = 3). Different letters represented statistically significant differences (two-way ANOVA, p<0.05).
(TIF)

S6 Fig HAT1 interacts with TPR3, but not with TPR1, TPR2 and TPR4. The ability of cells to grow on synthetic dropout medium lacking Leu, Trp, His, and Ade (-LWHA) suggested the interaction. AD, GAL4 activation domain. BD, GAL4 DNA binding domain.
(TIF)

S7 Fig The effects of TPL on HAT1 transcriptional repression activities in protoplasts. (A) A diagrammatical map of pGreenII-0800-LUC transient expression vector. REN, Renilla luciferase; LUC, firefly luciferase.
(B) Effects of TPL on HAT1 transcriptional repression activities of *DFR* promoters in *myb75-c* protoplasts. Error bars denote ± SD (n = 3). Different letters represented statistically significant differences (two-way ANOVA, p<0.05).
(TIF)

S8 Fig MYB75 does no interact with HAT2, HAT3, ATHB2, and ATHB4 in yeast. MYB75 does not interact with HAT2, HAT3, ATHB2, and ATHB4 in the yeast two-hybrid system. The ability of cells to grow on synthetic dropout medium lacking Leu, Trp, His, and Ade (-LWHA) suggested the interaction. Interaction between HAT1 and MYB75 served as a positive control. AD, GAL4 activation domain. BD, GAL4 DNA binding domain.
(TIF)

S9 Fig Anthocyanin accumulation in stem-rosette junction in Col-0, *35S:HAT1 #13*, and *hat1* plants. (A) Anthocyanin accumulation in the stem-rosette junction (arrowheads). Compared with the Col-0, *35S*:*HAT1 #13* plants accumulated less purple pigment (arrowheads), while *hat1* plants showed more anthocyanin accumulation (arrowheads). Bar = 1 cm.
(B) Anthocyanin levels in extracts from stems in (A). The experiments were performed in biological triplicate (representing anthocyanin content measured from 15 stems of each genotype and treatment were pooled for one replicate). FW, fresh weight. Error bars denote ± SD (n = 3). The asterisks imply the levels of statistic significance at *P < 0.05 and **P < 0.01 (Student’s t-test).
(TIF)

S10 Fig Expression of *MYB75*, *HAT1*, and *DFR* in stems and senescent leaves. (A) qPCR analysis of *MYB75*, *HAT1*, and *DFR* transcript levels in the lower, middle, and upper part of the inflorescence stems. Expression levels were standardized to *ACTIN 8*, and results in the basal were set at 1. Error bars denote ± SD (n = 3).
(B) qPCR analysis of *MYB75*, *HAT1*, and *DFR* transcript levels in the young leaves and senescent leaves. Expression levels were standardized to *ACTIN 8*, and results in the young leaves were set at 1. Error bars denote ± SD (n = 3).
(TIF)

S11 Fig Expression pattern of *MYB75*, *HAT1*, and *DFR* in plants. Arabidopsis eFP browser at http://bbc.botany.utoronto.ca/efp/cgi-bin/efpWeb.cgi produced the expression pattern of *MYB75* (A), *HAT1* (B), and *DFR* (C) [80]. The color scale at the left bottom corner represents the absolute transcript levels of gene: yellow means lower expression levels while red indicated higher.
(TIF)

S12 Fig Loss of *MYBL2* does not affect HAT1-repressed anthocyanin accumulation and histone H3 acetylation. (A) 14-day-old Arabidopsis seedlings of Col-0, *35S*:*HAT1 #13 mybl2* and *mybl2* grown on plates under different conditions. Bars = 0.5 cm.
(B) Anthocyanin levels in extracts from seedlings in (A). The experiments were performed in biological triplicate (representing anthocyanin content measured from 15 plants of each genotype and treatment were pooled for one replicate). FW, fresh weight. Error bars denote ± SD (n = 3). Different letters represented statistically significant differences (two-way ANOVA, p<0.05).
(C-E) ChIP-qPCR determines the histone H3 acetylation levels in the transcription start sites(TSSs) of *DFR*, *LDOX*, and *UF3GT* in Col-0, *35S*:*HAT1 #13 mybl2* and *mybl2* under different conditions. The histone H3 acetylation levels were standardized to *ACTIN 7*. and results in the Col-0 under control conditions were set at 1. Error bars denote ± SD (n = 3). Different letters represented statistically significant differences (two-way ANOVA, p<0.05).
(TIF)

S13 Fig Panorama of seedlings grown on plate for different genotype under different conditions. (A) Col-0, *35S*:*HAT1 #11*, *35S*:*HAT1 #13*, and *hat1* grown on plate under different conditions.
(B) *pap1-D*, *35S*:*HAT1 #13 pap1-D*, *myb75-c*, and *myb75-c hat1* grown on plate under different conditions.
(C) Col-0, *35S*:*HAT1 #13*, *35S*:*HAT1mEAR1 #6*, and *35S*:*HAT1mEAR1 #10* grown on plate under different conditions.
(D) *35S*:*MYB75*, *35S*:*HAT1 #13 35S*:*MYB75*, *35S*:*MYB75-HAT1N #2*, and *35S*:*MYB75-HAT1N #9* grown on plate under different conditions.
(TIF)

S14 Fig Confirmation of YFP expression in BiFC assays. (A) Expression levels of nYFP and cYFP in Fig 2C was monitored using RT-PCR. Shown are RT-PCR products after 26 cycles with nYFP and cYFP gene-specific or *Actin* gene-specific primers as a loading control.
(B) Expression levels of nYFP and cYFP in S4 Fig was monitored using RT-PCR. Shown are RT-PCR products after 26 cycles with nYFP and cYFP gene-specific or *Actin* gene-specific primers as a loading control.
(TIF)

S15 Fig Confirmation of *HAT1*, *HAT1mEAR1*, *MYB75* and *MYB75-HAT1N* in different genotype. (A) Transcript abundance of *HAT1* and *HAT1mEAR1* in different genotype was monitored using RT-PCR. Shown are RT-PCR products after 28 cycles with *HAT1* gene-specific or *ACTIN 8* gene-specific primers as a loading control.
(B) HAT1 and HAT1mEAR1 protein levels in the transgenic lines were detected by immunoblot analysis using an anti-GFP antibody. Actin was used as a loading control.
(C) Transcript abundance of *MYB75* and *MYB75-HAT1N* in different genotype was monitored using RT-PCR. Shown are RT-PCR products after 28 cycles with *MYB75* gene-specific, *MYB75-HAT1N* gene-specific, or *ACTIN 8* gene-specific primers as a loading control.
(TIF)

S16 Fig Full scan images of Western blots present in this study. (TIF)

S1 Table Primer sequences used in this study. (DOCX)

S2 Table Underlying data for Figs 1B, 1C–1E, 1F, 2C, 3B, 3C, 3D, 3E, 4B, 4C, 4D, 4E, 5D, 5F, 5G–5I, 6B, 6C, 6D–6F, 7B, 7C, 7D–7F, 8A–8C and 8D–8F. (XLSX)

S3 Table Underlying data for S1B, S2B, S2C, S2D, S5B, S5D, S7B, 9B, 10A, 10B, 12B and 12C–12E Figs. (XLSX)
