# Supplementary figures and images for: Regulation of anthocyanin accumulation via MYB75/HAT1/TPL-mediated transcriptional repression
Source: PLoS Genet. 2019 Mar 15;15(3):e1007993. doi: 10.1371/journal.pgen.1007993 (PMC6443190; doi:10.1371/journal.pgen.1007993)

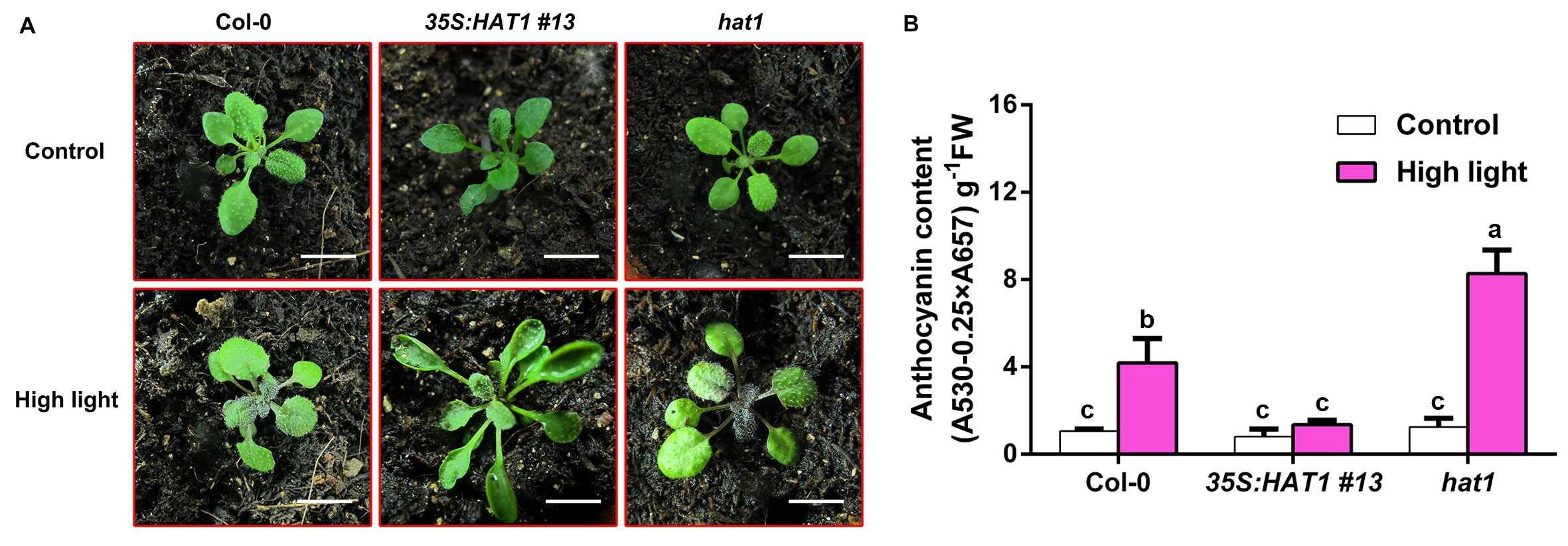

Supplement: S1 Fig — (A) 3-week-old Arabidopsis plants of Col-0, 35S:HAT1 #13, and hat1 grown in soil under different conditions. Bars = 1 cm. (B) Anthocyanin levels in extracts from seedlings in (A). The experiments were performed in biological triplicate (representing anthocyanin content measured from 15 plants of each genotype and treatment were pooled for one replicate). FW, fresh weight. Error bars denote ± SD (n = 3). Different letters represented statistically significant differences (two-way ANOVA, p<0.05). (TIF) [file pgen.1007993.s001.tif]

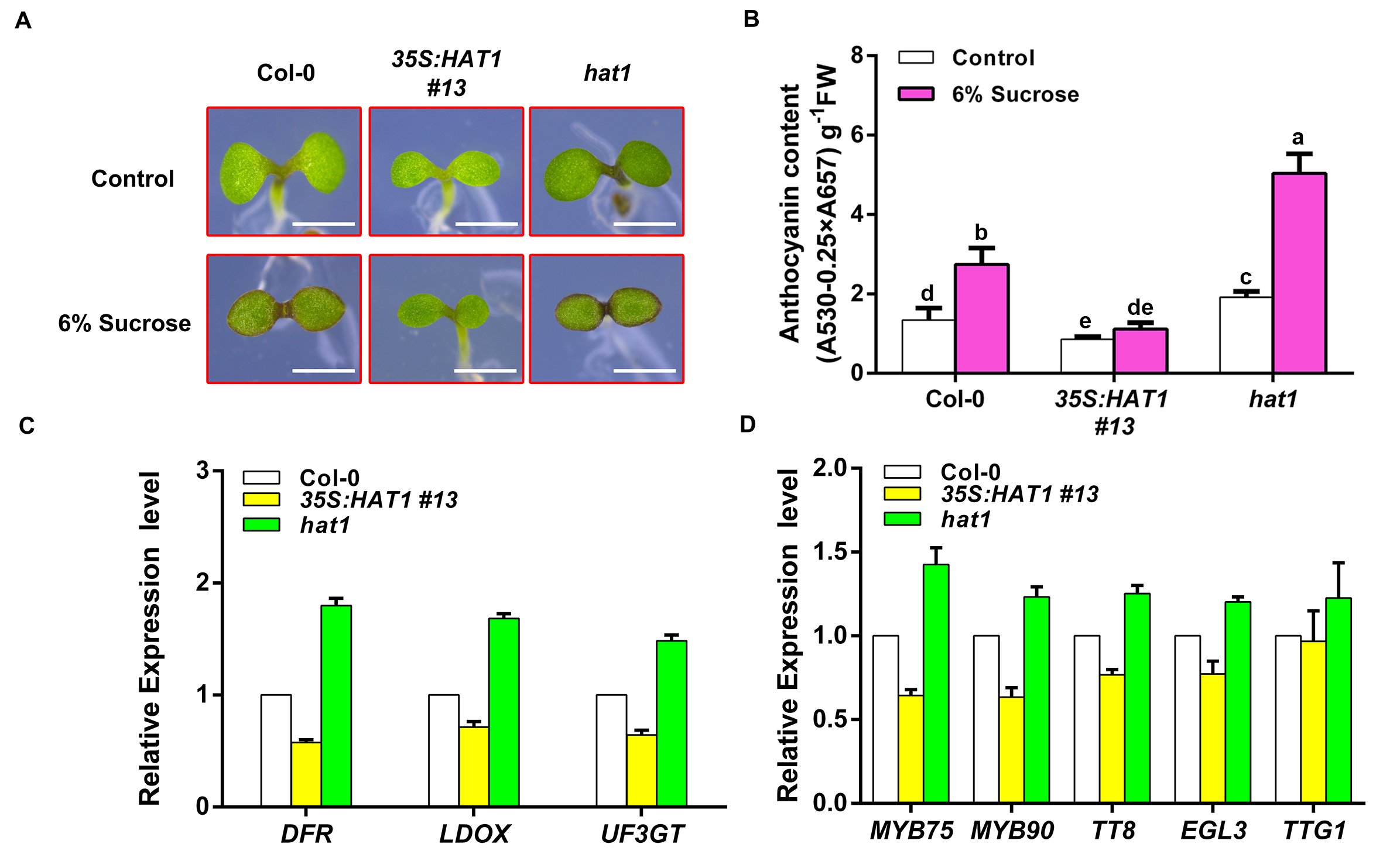

Supplement: S2 Fig — (A) 5-day-old Arabidopsis plants of Col-0, 35S:HAT1 #11, 35S:HAT1 #13, and hat1 grown in 1/2 MS media under control or 6% sucrose conditions. Bars = 1 mm. (B) Anthocyanin levels in extracts from seedlings in (A). The experiments were performed in biological triplicate (representing anthocyanin content measured from 15 plants of each genotype and treatment were pooled for one replicate). FW, fresh weight. Error bars denote ± SD (n = 3). Different letters represented statistically significant differences (two-way ANOVA, p<0.05). (C) qPCR analysis of DFR, LDOX and UF3GT expression levels in 3-day-old seedlings grown on 1/2 MS media. Expression levels were standardized to ACTIN 8, and results of Col-0 were set at 1. Error bars denote ± SD (n = 3). (D) qPCR analysis of anthocyanin regulatory genes transcript levels in 3-day-old seedlings grown on 1/2 MS media. Expression levels were standardized to ACTIN 8, and results of Col-0 under control conditions were set at 1. Error bars denote ± SD (n = 3). (TIF) [file pgen.1007993.s002.tif]

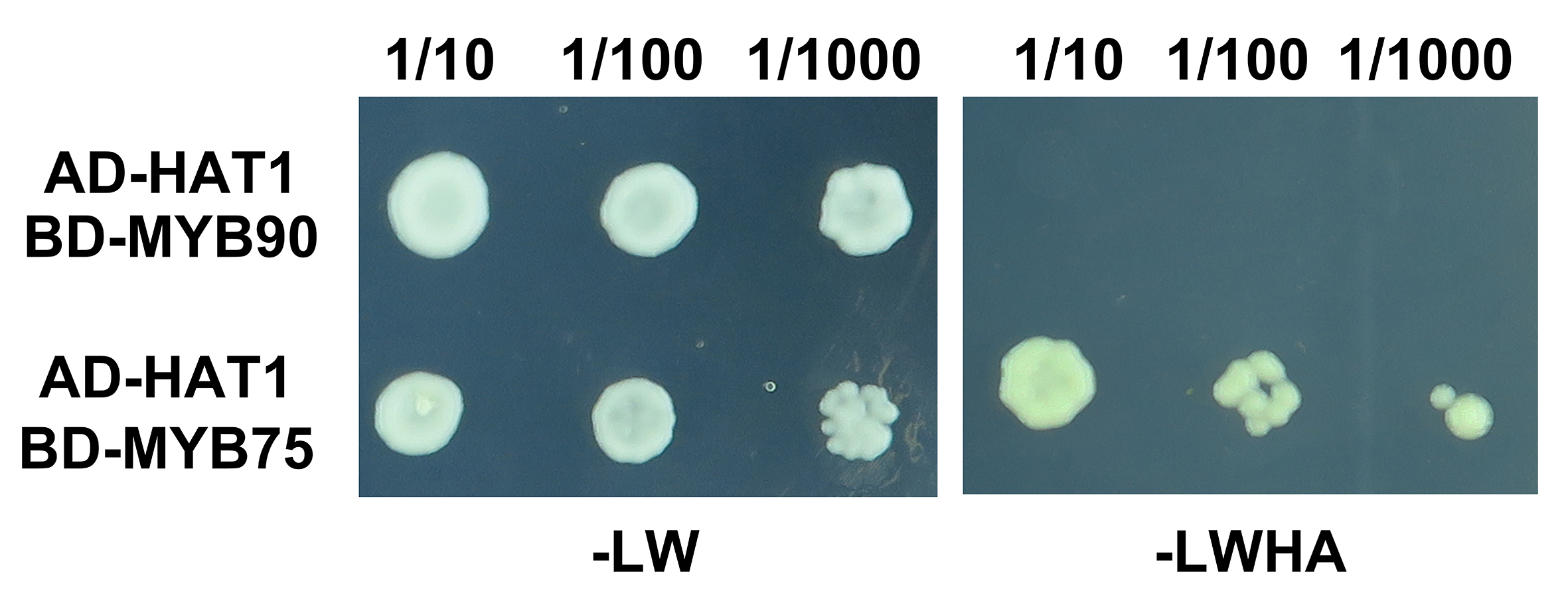

Supplement: S3 Fig — HAT1 does not interact with MYB90 in yeast. The ability of cells to grow on synthetic dropout medium lacking Leu, Trp, His, and Ade (-LWHA) suggested the interaction. Interaction between HAT1 and MYB75 served as a positive control. AD, GAL4 activation domain. BD, GAL4 DNA binding domain. (TIF) [file pgen.1007993.s003.tif]

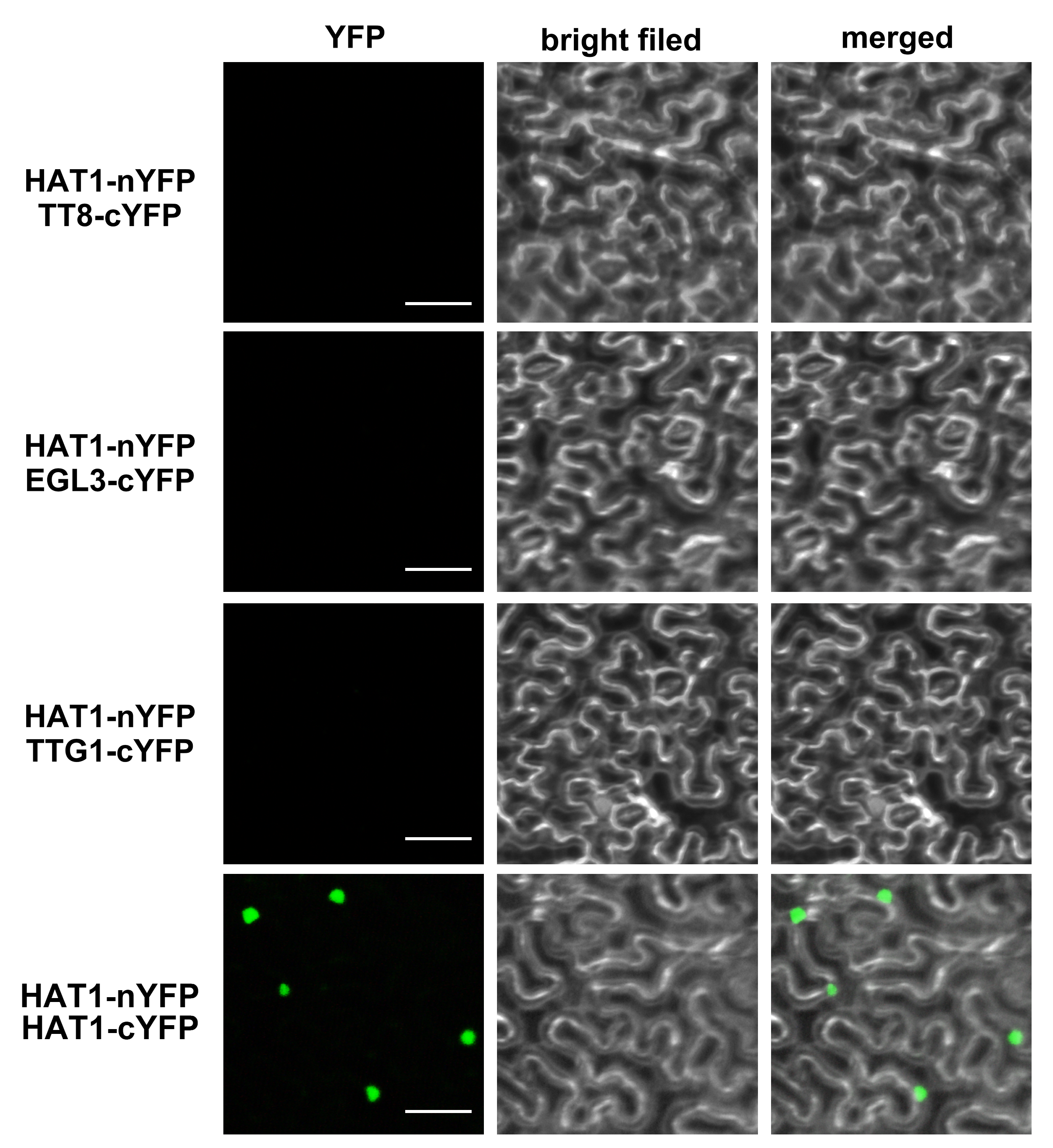

Supplement: S4 Fig — No signals of YFP fluorescence were observed in tobacco leaves when co-expression of HAT1-nYFP and TT8-cYFP, HAT1-nYFP and EGL3-cYFP, HAT1-nYFP and TTG1-cYFP. Co-expression of HAT1-nYFP and HAT1-cYFP served as transformation control. Bars = 50 μm. (TIF) [file pgen.1007993.s004.tif]

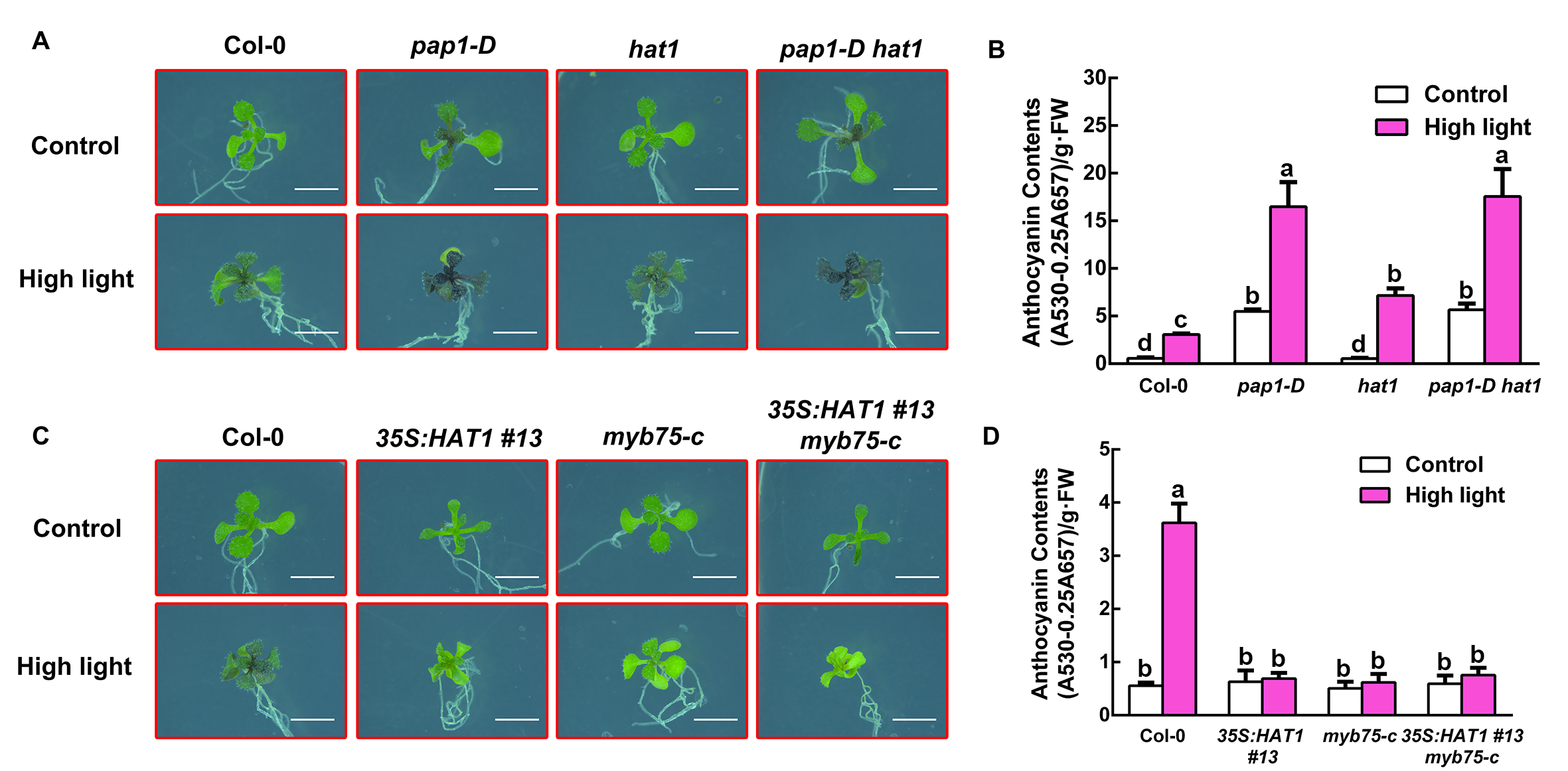

Supplement: S5 Fig — (A) 14-day-old Arabidopsis seedlings of Col-0, pap1-D, hat1, pap1-D hat1 grown on plates under different conditions. Bars = 0.5 cm. (B) Anthocyanin levels in extracts from seedlings in (A). The experiments were performed in biological triplicate (representing anthocyanin content measured from 15 plants of each genotype and treatment were pooled for one replicate). FW, fresh weight. Error bars denote ± SD (n = 3). Different letters represented statistically significant differences (two-way ANOVA, p<0.05). (C) 14-day-old Arabidopsis seedlings of Col-0, 35S:HAT1 #13, myb75-c, 35S:HAT1 #13 myb75-c grown on plates under different conditions. Bars = 0.5 cm. (D) Anthocyanin levels in extracts from seedlings in (C). The experiments were performed in biological triplicate (representing anthocyanin content measured from 15 plants of each genotype and treatment were pooled for one replicate). FW, fresh weight. Error bars denote ± SD (n = 3). Different letters represented statistically significant differences (two-way ANOVA, p<0.05). (TIF) [file pgen.1007993.s005.tif]

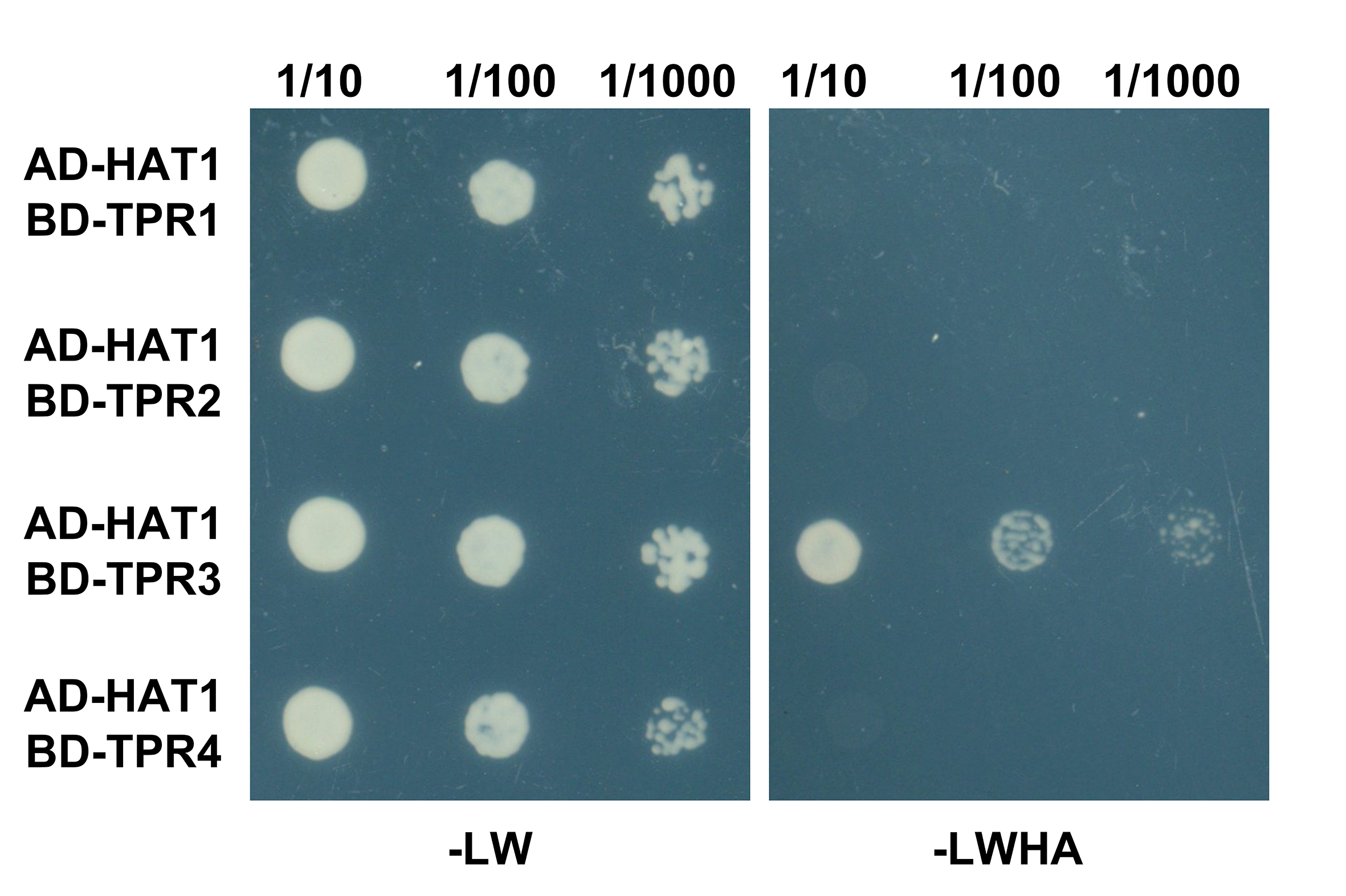

Supplement: S6 Fig — The ability of cells to grow on synthetic dropout medium lacking Leu, Trp, His, and Ade (-LWHA) suggested the interaction. AD, GAL4 activation domain. BD, GAL4 DNA binding domain. (TIF) [file pgen.1007993.s006.tif]

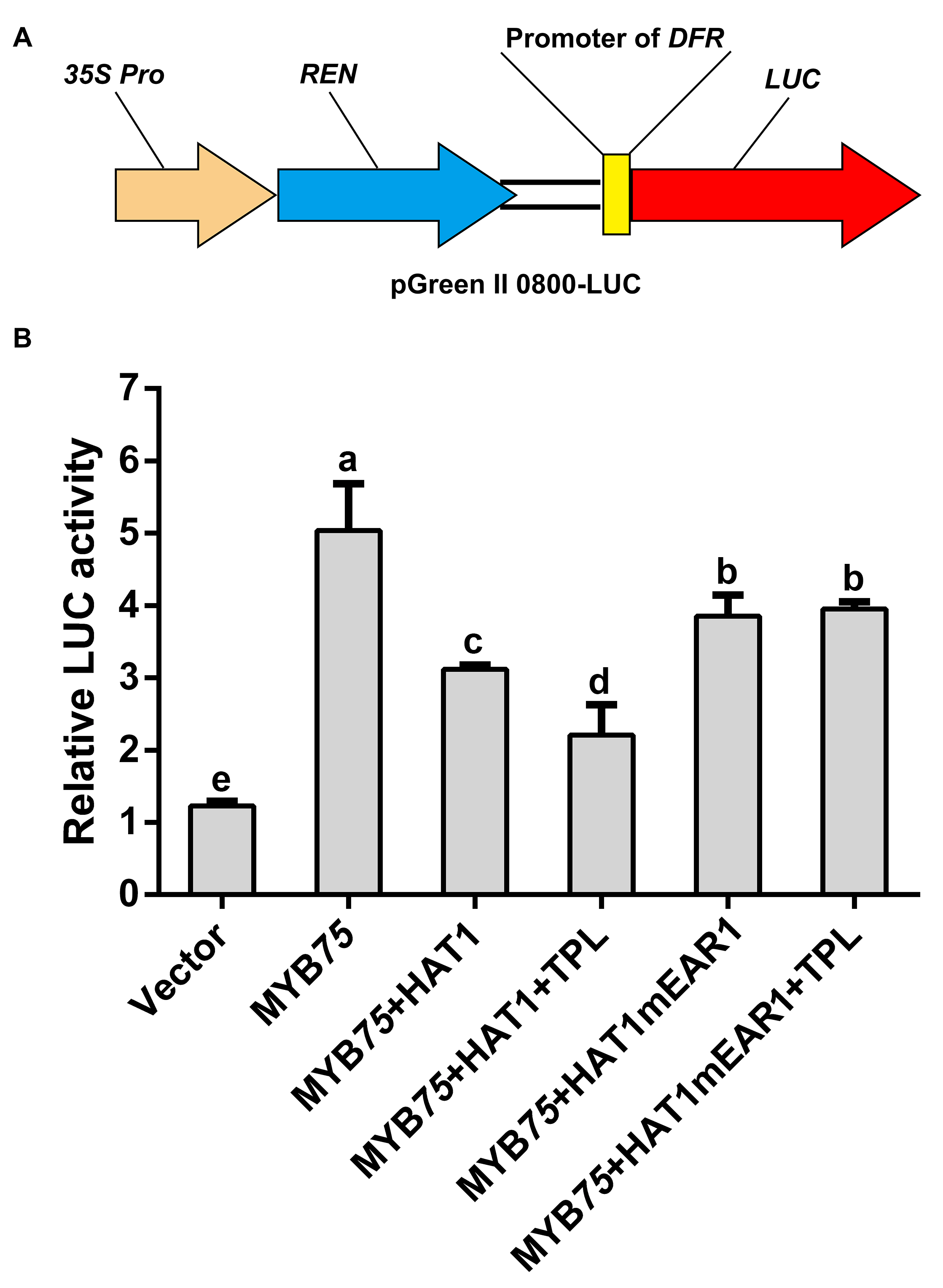

Supplement: S7 Fig — (A) A diagrammatical map of pGreenII-0800-LUC transient expression vector. REN, Renilla luciferase; LUC, firefly luciferase. (B) Effects of TPL on HAT1 transcriptional repression activities of DFR promoters in myb75-c protoplasts. Error bars denote ± SD (n = 3). Different letters represented statistically significant differences (two-way ANOVA, p<0.05). (TIF) [file pgen.1007993.s007.tif]

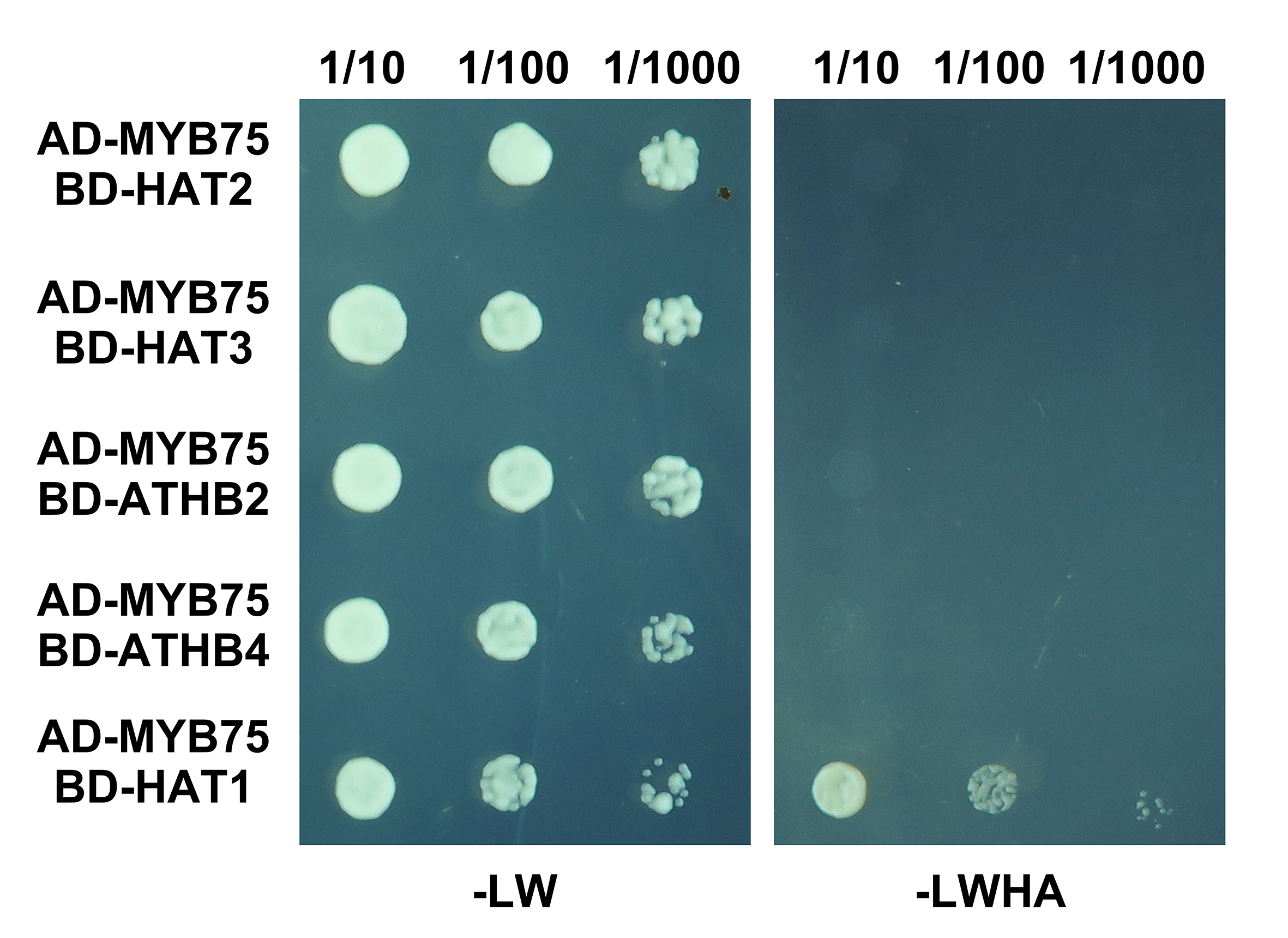

Supplement: S8 Fig — MYB75 does not interact with HAT2, HAT3, ATHB2, and ATHB4 in the yeast two-hybrid system. The ability of cells to grow on synthetic dropout medium lacking Leu, Trp, His, and Ade (-LWHA) suggested the interaction. Interaction between HAT1 and MYB75 served as a positive control. AD, GAL4 activation domain. BD, GAL4 DNA binding domain. (TIF) [file pgen.1007993.s008.tif]

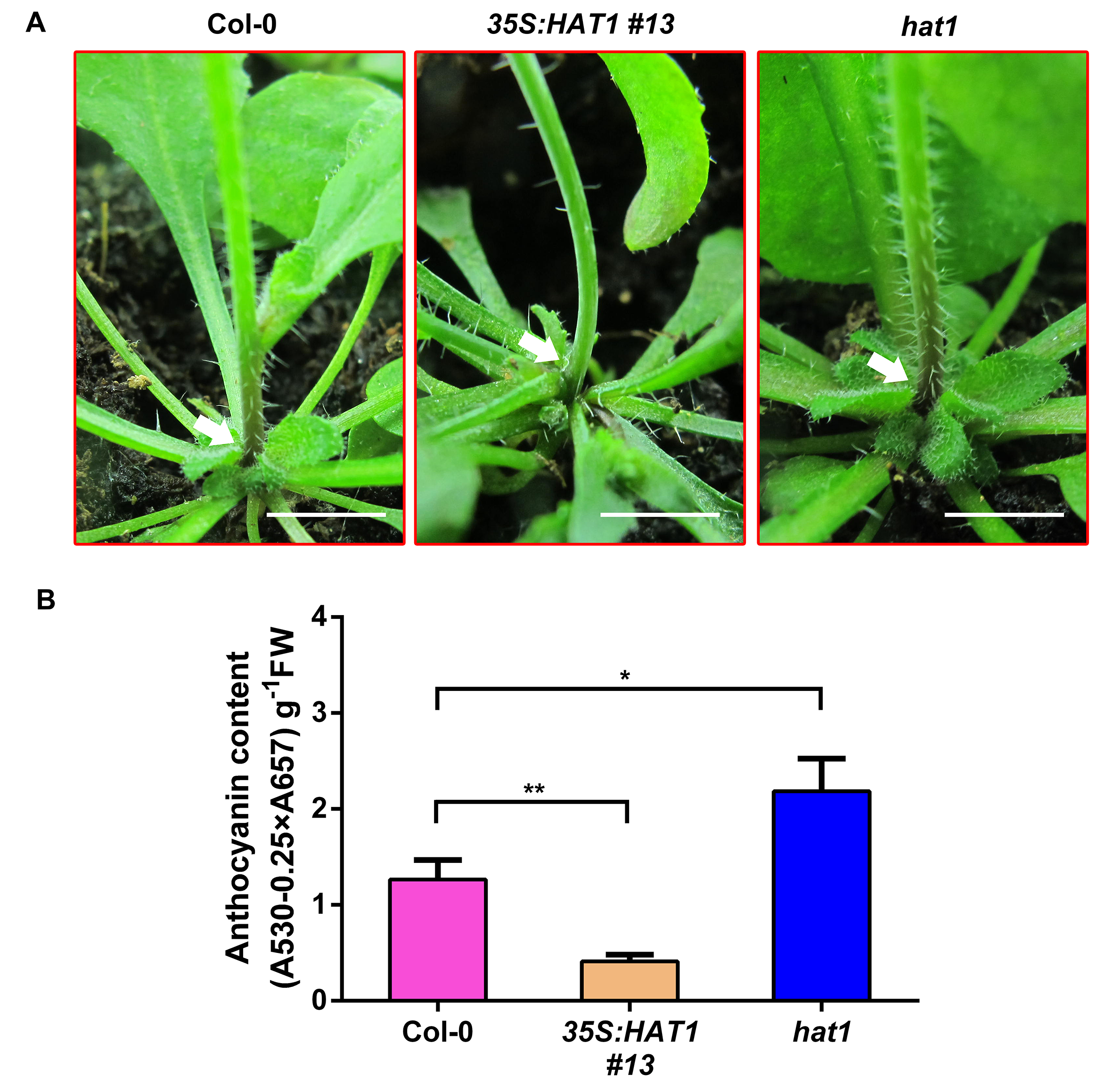

Supplement: S9 Fig — (A) Anthocyanin accumulation in the stem-rosette junction (arrowheads). Compared with the Col-0, 35S:HAT1 #13 plants accumulated less purple pigment (arrowheads), while hat1 plants showed more anthocyanin accumulation (arrowheads). Bar = 1 cm. (B) Anthocyanin levels in extracts from stems in (A). The experiments were performed in biological triplicate (representing anthocyanin content measured from 15 stems of each genotype and treatment were pooled for one replicate). FW, fresh weight. Error bars denote ± SD (n = 3). The asterisks imply the levels of statistic significance at *P < 0.05 and **P < 0.01 (Student’s t-test). (TIF) [file pgen.1007993.s009.tif]

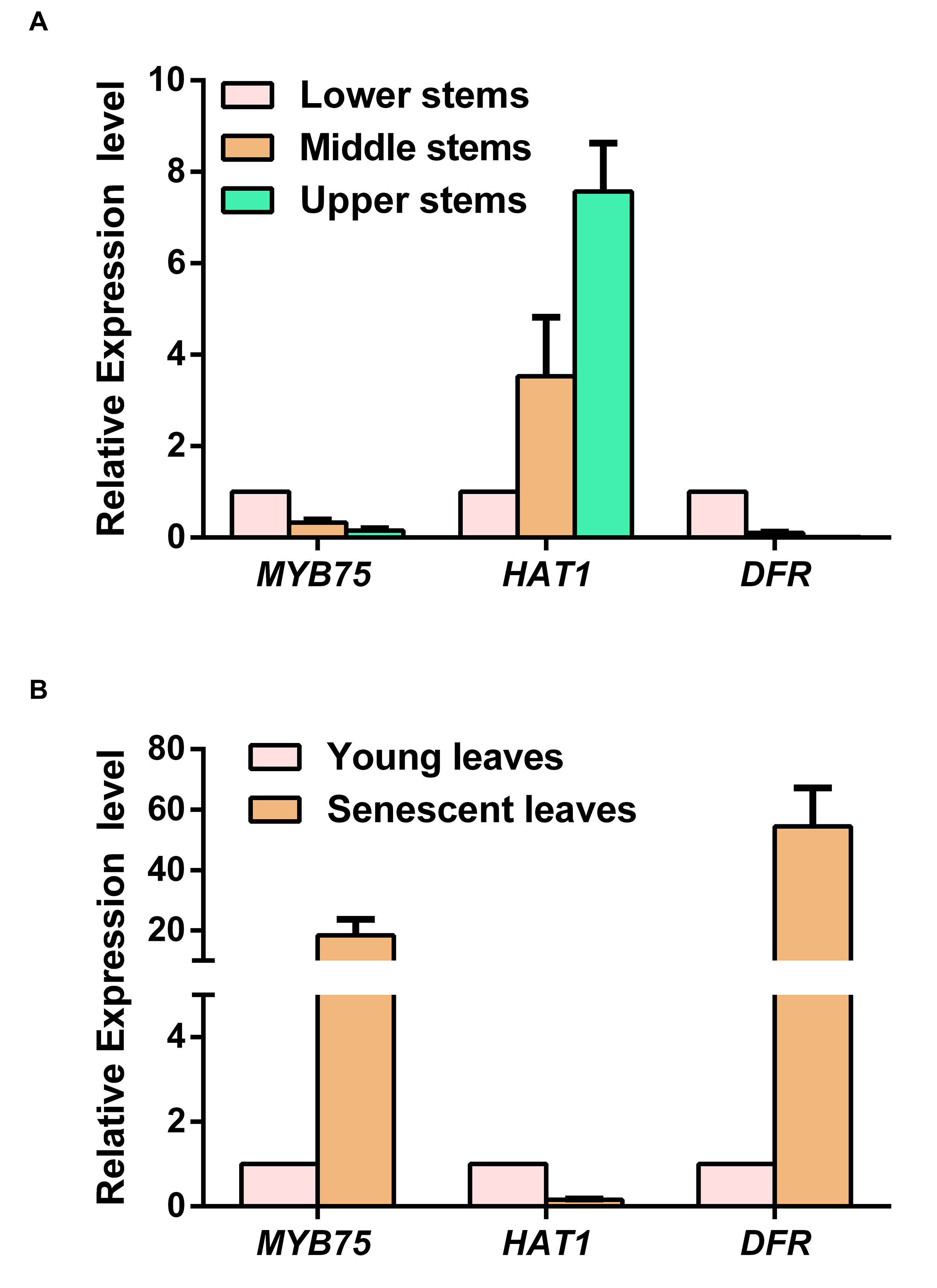

Supplement: S10 Fig — (A) qPCR analysis of MYB75, HAT1, and DFR transcript levels in the lower, middle, and upper part of the inflorescence stems. Expression levels were standardized to ACTIN 8, and results in the basal were set at 1. Error bars denote ± SD (n = 3). (B) qPCR analysis of MYB75, HAT1, and DFR transcript levels in the young leaves and senescent leaves. Expression levels were standardized to ACTIN 8, and results in the young leaves were set at 1. Error bars denote ± SD (n = 3). (TIF) [file pgen.1007993.s010.tif]

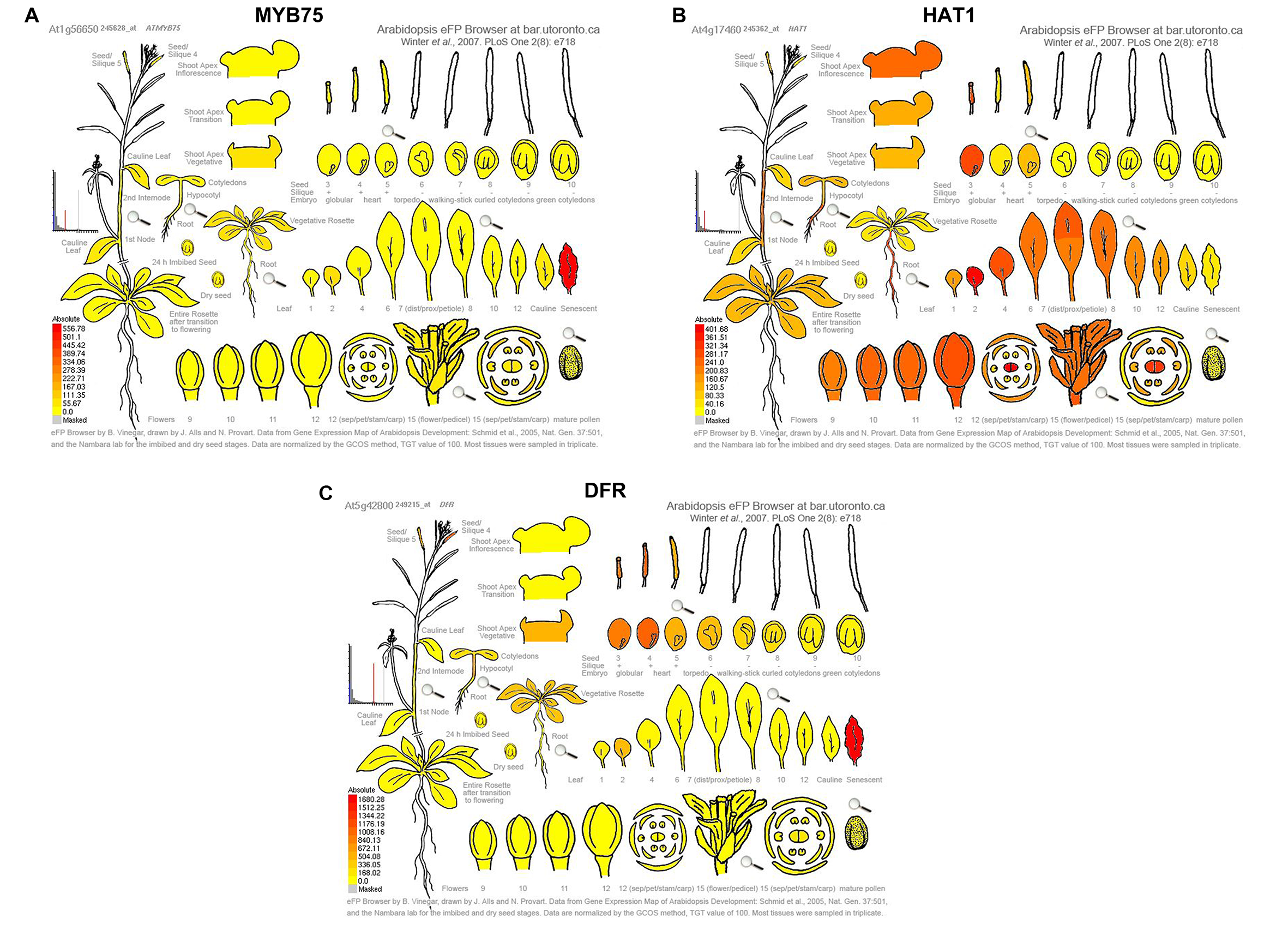

Supplement: S11 Fig — Arabidopsis eFP browser at http://bbc.botany.utoronto.ca/efp/cgi-bin/efpWeb.cgi produced the expression pattern of MYB75 (A), HAT1 (B), and DFR (C) [80]. The color scale at the left bottom corner represents the absolute transcript levels of gene: yellow means lower expression levels while red indicated higher. (TIF) [file pgen.1007993.s011.tif]

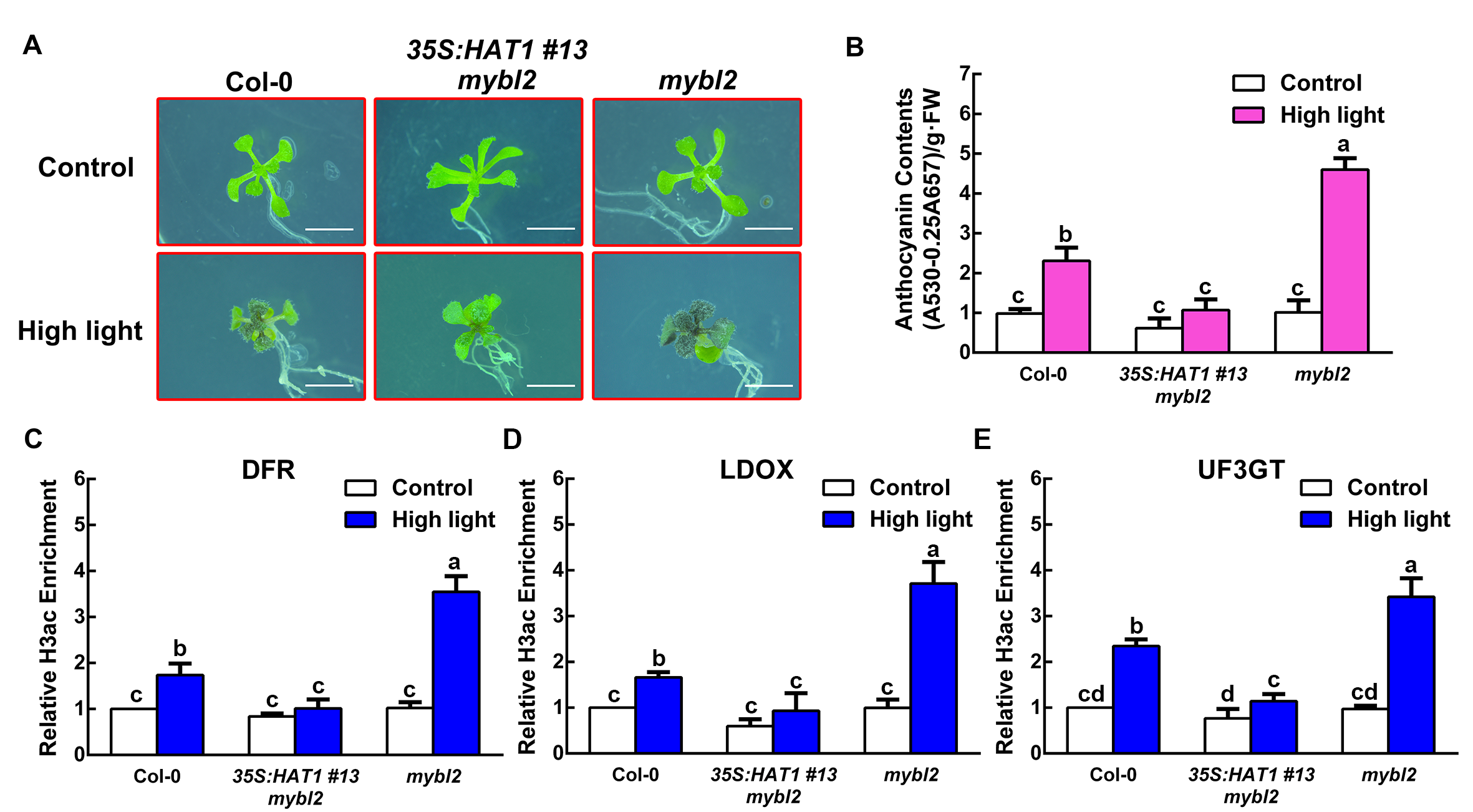

Supplement: S12 Fig — (A) 14-day-old Arabidopsis seedlings of Col-0, 35S:HAT1 #13 mybl2 and mybl2 grown on plates under different conditions. Bars = 0.5 cm. (B) Anthocyanin levels in extracts from seedlings in (A). The experiments were performed in biological triplicate (representing anthocyanin content measured from 15 plants of each genotype and treatment were pooled for one replicate). FW, fresh weight. Error bars denote ± SD (n = 3). Different letters represented statistically significant differences (two-way ANOVA, p<0.05). (C-E) ChIP-qPCR determines the histone H3 acetylation levels in the transcription start sites(TSSs) of DFR, LDOX, and UF3GT in Col-0, 35S:HAT1 #13 mybl2 and mybl2 under different conditions. The histone H3 acetylation levels were standardized to ACTIN 7. and results in the Col-0 under control conditions were set at 1. Error bars denote ± SD (n = 3). Different letters represented statistically significant differences (two-way ANOVA, p<0.05). (TIF) [file pgen.1007993.s012.tif]

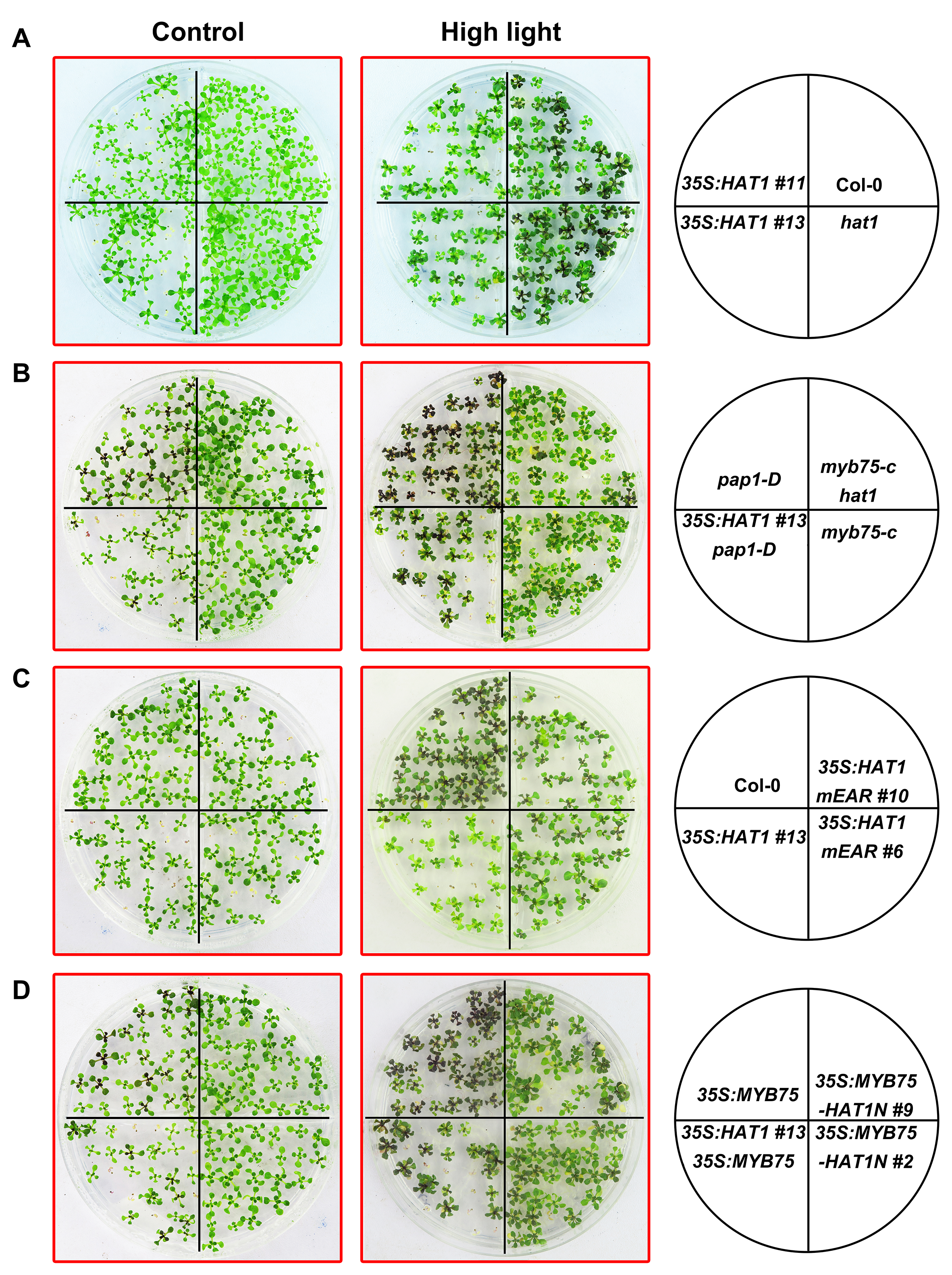

Supplement: S13 Fig — (A) Col-0, 35S:HAT1 #11, 35S:HAT1 #13, and hat1 grown on plate under different conditions. (B) pap1-D, 35S:HAT1 #13 pap1-D, myb75-c, and myb75-c hat1 grown on plate under different conditions. (C) Col-0, 35S:HAT1 #13, 35S:HAT1mEAR1 #6, and 35S:HAT1mEAR1 #10 grown on plate under different conditions. (D) 35S:MYB75, 35S:HAT1 #13 35S:MYB75, 35S:MYB75-HAT1N #2, and 35S:MYB75-HAT1N #9 grown on plate under different conditions. (TIF) [file pgen.1007993.s013.tif]

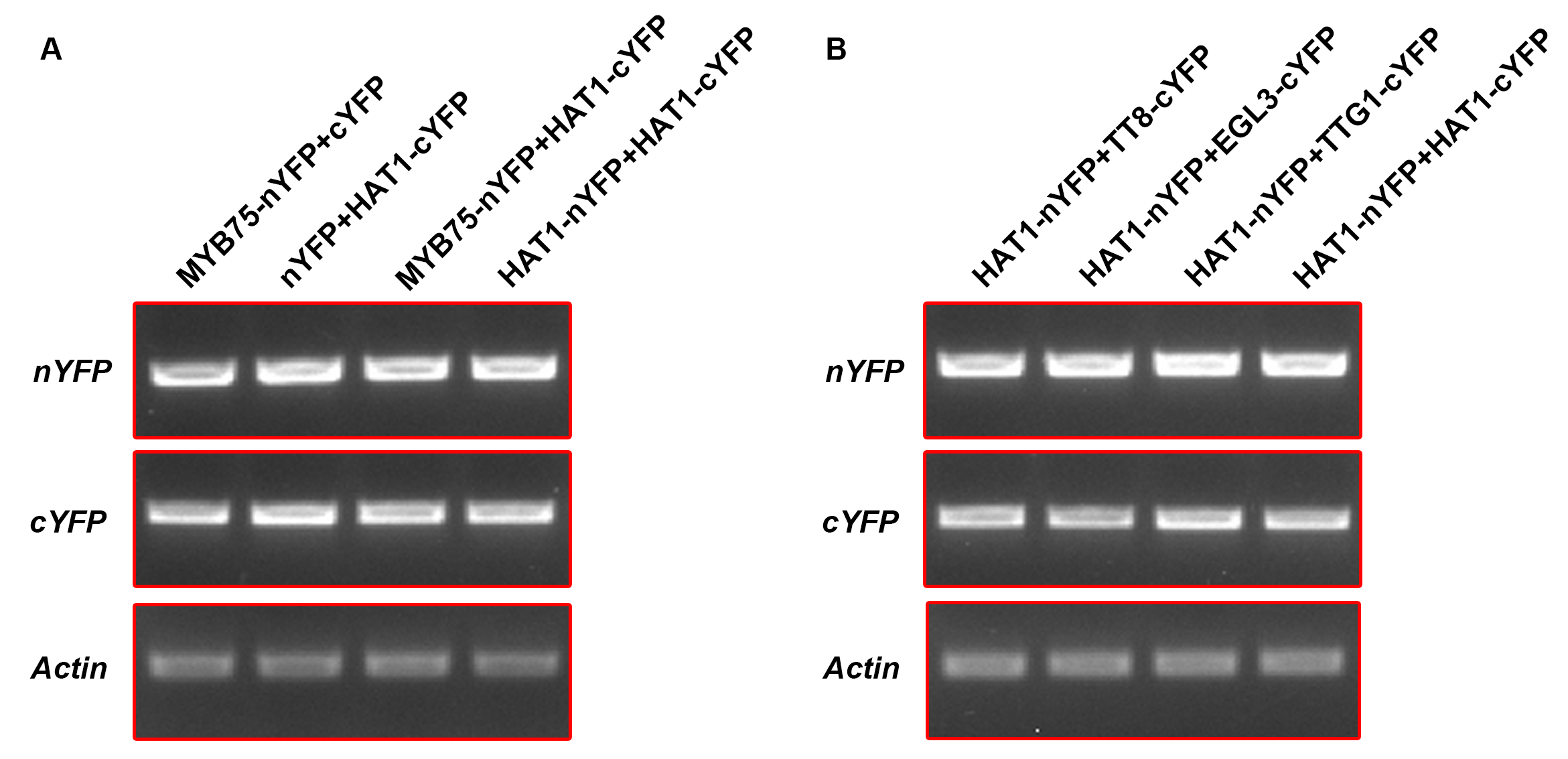

Supplement: S14 Fig — (A) Expression levels of nYFP and cYFP in Fig 2C was monitored using RT-PCR. Shown are RT-PCR products after 26 cycles with nYFP and cYFP gene-specific or Actin gene-specific primers as a loading control. (B) Expression levels of nYFP and cYFP in S4 Fig was monitored using RT-PCR. Shown are RT-PCR products after 26 cycles with nYFP and cYFP gene-specific or Actin gene-specific primers as a loading control. (TIF) [file pgen.1007993.s014.tif]

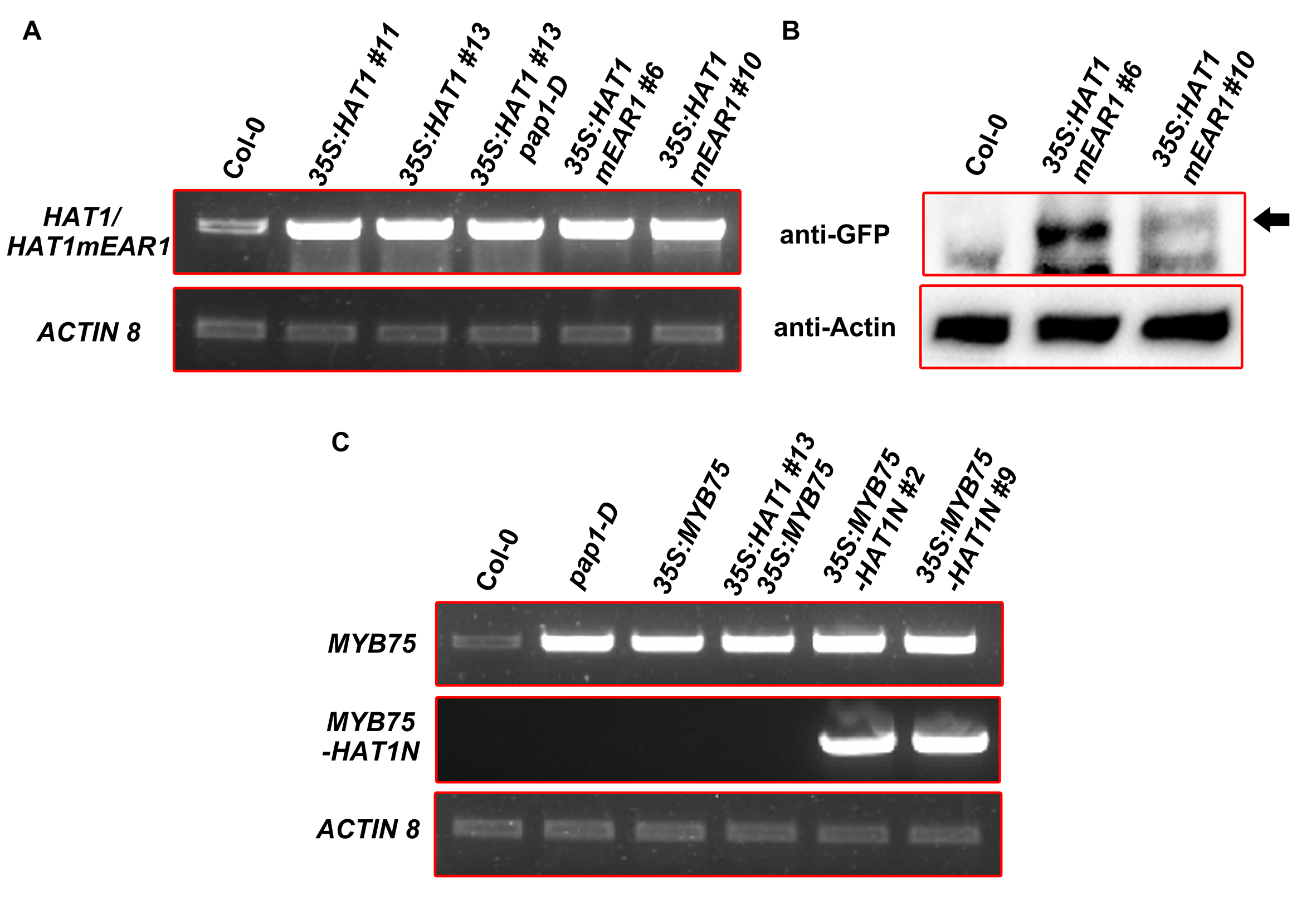

Supplement: S15 Fig — (A) Transcript abundance of HAT1 and HAT1mEAR1 in different genotype was monitored using RT-PCR. Shown are RT-PCR products after 28 cycles with HAT1 gene-specific or ACTIN 8 gene-specific primers as a loading control. (B) HAT1 and HAT1mEAR1 protein levels in the transgenic lines were detected by immunoblot analysis using an anti-GFP antibody. Actin was used as a loading control. (C) Transcript abundance of MYB75 and MYB75-HAT1N in different genotype was monitored using RT-PCR. Shown are RT-PCR products after 28 cycles with MYB75 gene-specific, MYB75-HAT1N gene-specific, or ACTIN 8 gene-specific primers as a loading control. (TIF) [file pgen.1007993.s015.tif]

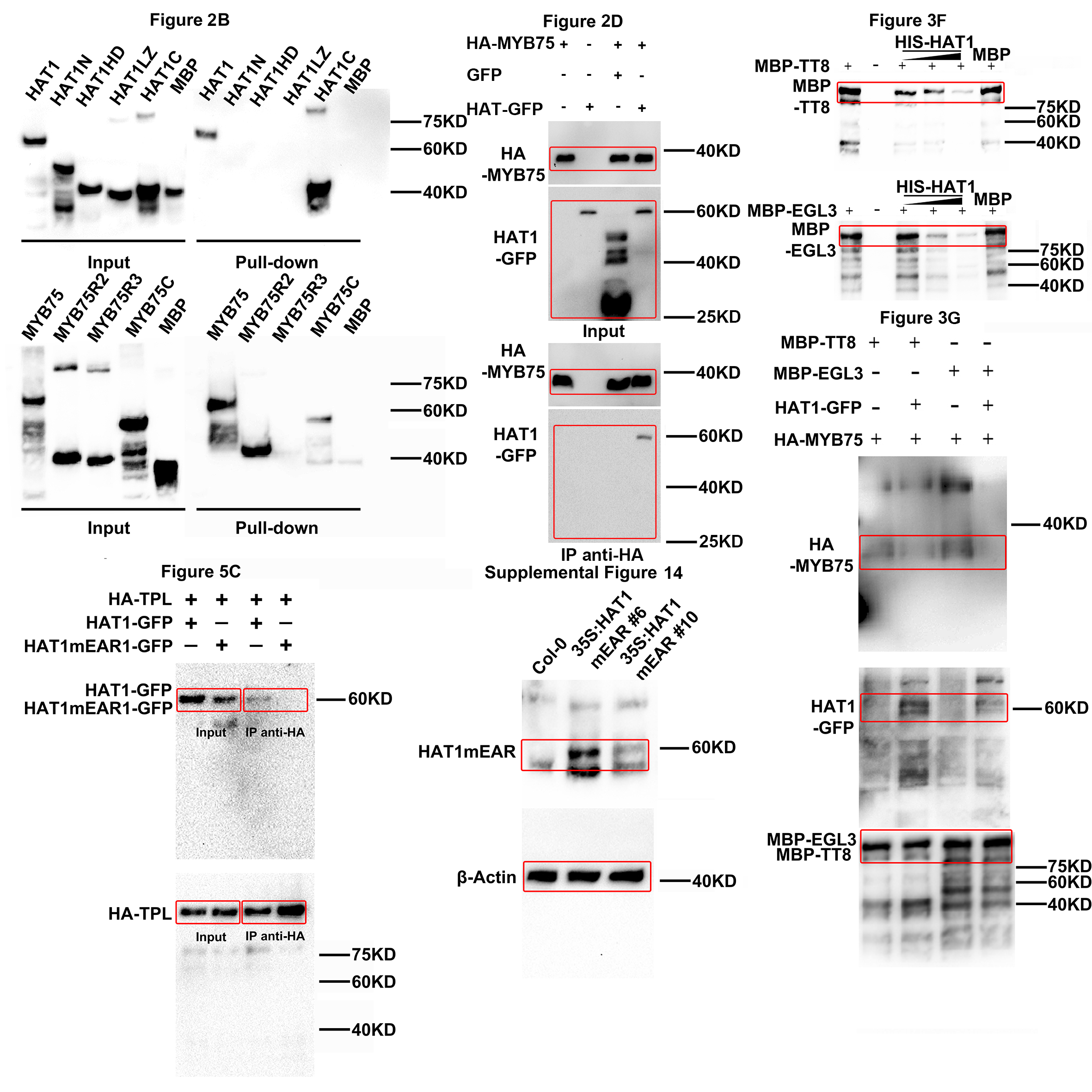

Supplement: S16 Fig — (TIF) [file pgen.1007993.s016.tif]
