# Supplementary material for: Regulation of anthocyanin accumulation via MYB75/HAT1/TPL-mediated transcriptional repression
Source: PLoS Genet. 2019 Mar 15;15(3):e1007993. doi: 10.1371/journal.pgen.1007993 (PMC6443190; doi:10.1371/journal.pgen.1007993)
Supplement: S1 Table — (DOCX) [file pgen.1007993.s017.docx]

| Primer Names | Primer Sequences (5’-3’) | Purpose |
| --- | --- | --- |
| Yeast-MYB75-F | CCGGAATTCATGGAGGGTTCGTCCAAAGG | Y2H |
| Yeast-MYB75-R | CGCGGATCCCTAATCAAATTTCACAGTCT | Y2H |
| Yeast-MYB90-F | CCGGAATTCATGGAGGGTTCGTCCAAAGG | Y2H |
| Yeast-MYB90-R | CGCGGATCCCTAATCAAGTTCAACAGTCT | Y2H |
| Yeast-HAT1-F | CCGGAATTCATGATGATGGGTAAAGAGGA | Y2H |
| Yeast-HAT1-R | CGCGGATCCTTAAGACCTAGGACGCATCA | Y2H |
| Yeast-HAT1mEAR1-F | ATGGGTAAAGAGGATGCGGGTGCAAGTGCTAGCTTGGGATTTGCA | Y2H |
| Yeast-HAT1mEAR1-R | TGCAAATCCCAAGCTAGCACTTGCACCCGCATCCTCTTTACCCAT | Y2H |
| Yeast-HAT1mEAR2-F | GCACAAAACCATCCTGCCCAGGCAAATGCTAAACCCACTTCTTCA | Y2H |
| Yeast-HAT1mEAR2-R | TGAAGAAGTGGGTTTAGCATTTGCCTGGGCAGGATGGTTTTGTGC | Y2H |
| Yeast-TPL-F | TCCCCCGGGGGATGTCTTCTCTTAGTAGAGA | Y2H |
| Yeast-TPL-R | ACGCGTCGACTCATCTCTGAGGCTGATCAG | Y2H |
| Yeast-TPR1-F | CCGGAATTCATGTCTTCTCTGAGCAGAGA | Y2H |
| Yeast-TPR1-R | CGCGGATCCTCATCTCTGAGGCTGGTCAG | Y2H |
| Yeast-TPR2-F | CCGGAATTCATGTCGTCTTTGAGCAGAGA | Y2H |
| Yeast-TPR2-R | ACGCGTCGACTTACCTTTGAATCTGATCCG | Y2H |
| Yeast-TPR3-F | CGCGGATCCGTATGTCGTCGTTGAGTCGAGA | Y2H |
| Yeast-TPR3-R | AACTGCAGTCATCTTTGTAACTGTTCTG | Y2H |
| Yeast-TPR4-F | GGAATTCCATATGATGTCGTCACTCAGCAGAGA | Y2H |
| Yeast-TPR4-R | CGCGGATCCCTACGAATCACTCGGTTGTT | Y2H |
| Yeast-HAT2-F | CCGGAATTCATGATGATGGGCAAAGAAGA | Y2H |
| Yeast-HAT2-R | CGCGGATCCTCACGATCGTGGACGCAAGG | Y2H |
| Yeast-HAT3-F | CGCGGATCCGTATGAGTGAAAGAGATGATGG | Y2H |
| Yeast-HAT3-R | AACTGCAGCTAATGAGAACCAGCAGCAG | Y2H |
| Yeast-ATHB2-F | CCGGAATTCATGATGTTCGAGAAAGACGA | Y2H |
| Yeast-ATHB2-R | CGCGGATCCTTAGGACCTAGGACGAAGAG | Y2H |
| Yeast-ATHB4-F | CCGGAATTCATGGGGGAAAGAGATGATGG | Y2H |
| Yeast-ATHB4-R | CGCGGATCCCTAGCGACCTGATTTTTGCT | Y2H |
| BiFC-MYB75-F | CGCGGATCCATGGAGGGTTCGTCCAAAGG | BiFC |
| BiFC-MYB75-R | ACGCGTCGACATCAAATTTCACAGTCTCTCCA | BiFC |
| BiFC-TT8-F | CGCGGATCCATGGATGAATCAAGTATTAT | BiFC |
| BiFC-TT8-R | ACGCGTCGACTAGATTAGTATCATGTATTATG | BiFC |
| BiFC-EGL3-F | CGCGGATCCATGGCAACCGGAGAAAAC | BiFC |
| BiFC-EGL3-R | ACGCGTCGACACATATCCATGCAACCCTTT | BiFC |
| BiFC-TTG1-F | TGCTCTAGAATGGATAATTCAGCTCCAGA | BiFC |
| BiFC-TTG1-R | ACGCGTCGACAACTCTAAGGAGCTGCATTT | BiFC |
| MYB75-F | TTCGTCCAAAGGGCTGC | BiFC |
| MYB75-R | TCTACAACTTTTCCTGCACCG | BiFC |
| TT8-F | CGCTAAGAGTGCTAAAATTCAGAC | BiFC |
| TT8-R | TTCGTCTTGCAGTGGTCATAG | BiFC |
| EGL3-F | TCTTTCGTCTTCAACATCGGTG | BiFC |
| EGL3-R | GCGGTTTCAGCGTTACAAAG | BiFC |
| *Nicotiana* Actin-F | TAACCCAAAGGCTAATCGTG | BiFC |
| *Nicotiana* Actin-R | GTAGTCTCGTGGATTCCTGC | BiFC |
| MBP-MYB75-F | CTAGTCTAGAATGGAGGGTTCGTCCAAAGG | Pull down |
| MBP-MYB75-R | CCCATCGATCTAATCAAATTTCACAGTCT | Pull down |
| MBP-MYB75(R2)-R | CCCATCGATCTAACTTGGCTTCAAATAGTTCA | Pull down |
| MBP-MYB75(R3)-F | CTAGTCTAGAATCAAGAGAGGAAAACTTAG | Pull down |
| MBP-MYB75(R3)-R | CCCATCGATCTATTTCTTACTCAGATGAGTGT | Pull down |
| MBP-MYB75(CT)-F | CTAGTCTAGACATGAACCGTGTTGTAAGAT | Pull down |
| HIS-MYB75-F | CGCGGATCCATGGAGGGTTCGTCCAAAGG | Pull down |
| HIS-MYB75-R | ACGCGTCGACCTAATCAAATTTCACAGTCT | Pull down |
| HIS-HAT1-F | CGCGGATCCATGATGATGG GTAAAGAGGA | Pull down |
| HIS-HAT1-R | ACGCGTCGACTTAAGACCTAGGACGCATCA | Pull down |
| MBP-TT8-F | CGCGGATCCATGGATGAATCAAGTATTAT | Pull down |
| MBP-TT8-R | ACGCGTCGACCTATAGATTAGTATCATGTATT | Pull down |
| MBP-EGL3-F | CGCGGATCCATGGCAACCGGAGAAAAC | Pull down |
| MBP-EGL3-R | ACGCGTCGACTTAACATATCCATGCAACCC | Pull down |
| HA-MYB75-F | ACGCGTCGACATGGAGGGTTCGTCCAAAGG | CoIP, LUC |
| HA-MYB75-R | CGGGGTACCCTAATCAAATTTCACAGTCT | CoIP, LUC |
| HA-TPL-F | ACGCGTCGACATGTCTTCTCTTAGTAGAGA | CoIP, LUC |
| HA-TPL-R | CGGGGTACCTCATCTCTGAGGCTGATCAG | CoIP, LUC |
| HAT1ΔEAR1-GFP-F | CGCGGATCCGGATTTGCACAAAACCATCC | LUC |
| HAT1ΔEAR1-GFP-R | ACGCGTCGACTTAAGACCTAGGACGCATCA | LUC |
| HAT1ΔEAR2-GFP-F | CGCGGATCCAAACCCACTTCTTCACCAAT | LUC |
| HAT1ΔEAR2-GFP-R | ACGCGTCGACTTAAGACCTAGGACGCATCA | LUC |
| ProDFR-F | CCCAAGCTTGCTTTTCCAAGATTTATAAT | LUC |
| ProDFR-R | CGCGGATCCTTTTGTGGTTATATGATAGA | LUC |
| HAT1mEAR-GFP-F | CGCGGATCCATGATGATGGGTAAAGAGGA | Overexpression |
| HAT1mEAR-GFP-R | ACGCGTCGACAGACCTAGGACGCATCA | Overexpression |
| HA-MYB75-F | ACGCGTCGACATACCTTTTACAATTTGTTT | Overexpression |
| HA-MYB75-R | TGCTCTAGAATCAAATTTCACAGTCTCTC | Overexpression |
| HAT1N-F | TGCTCTAGAATGATGATGGGTAAAGAGGA | Overexpression |
| HAT1N-R | CGGGGTACCTTATCCACTCACTGTGCTCGAGA | Overexpression |
| DFR-F | GTGGTTACTTTGTTCGTGCC | qPCR |
| DFR-R | ACCGTCACATCCGTTTATGG | qPCR |
| LDOX-F | TGTCAAGAAAGCCGGAGAAG | qPCR |
| LDOX-R | AAGACACTTCGCGTACTCAC | qPCR |
| UF3GT-F | CAGCACGGAGAACAGATTTTG | qPCR |
| UF3GT-R | CTCACTTTCTCACCGATCTCAC | qPCR |
| ACTIN 8-F | GCTCCGTATTGCTCCTGAAG | qPCR |
| ACTIN 8-R | GAGGATAGCATGTGGAAGTGAG | qPCR |
| ChIP-DFR-F | TCTGACGTCTTACGATACAA | ChIP-qPCR |
| ChIP-DFR-R | TTCCAGTTTTCGCAAAGAAA | ChIP-qPCR |
| ChIP-LDOX-F | ATTGAAAACCAGTCAAATTG | ChIP-qPCR |
| ChIP-LDOX-R | GAAAAGGCCCAATAGAATTA | ChIP-qPCR |
| ChIP-UF3GT-F | TTGCTTAAAAAGGGGGCCGA | ChIP-qPCR |
| ChIP-UF3GT-R | GAGTGTCCTGATGAATGGTG | ChIP-qPCR |
| ChIP-ACTIN 7-F | CCATAGCATTGTCTCTCCCA | ChIP-qPCR |
| ChIP-ACTIN 7-R | CGGCAGCAGCTAACACTAAG | ChIP-qPCR |
